# Supplementary material for: Identification of a TNF-TNFR-like system in malaria vectors (Anopheles stephensi) likely to influence Plasmodium resistance
Source: Sci Rep. 2022 Nov 9;12:19079. doi: 10.1038/s41598-022-23780-y (PMC9646898; doi:10.1038/s41598-022-23780-y)
Supplement: Supplementary file 1 — Supplementary Information 1. [file 41598_2022_23780_MOESM1_ESM.pdf]

# **Identification of a TNF-TNFR-like system in malaria vectors (*Anopheles stephensi*) likely to influence *Plasmodium* resistance**

Subhashini Srinivasan[1,*], Chaitali Ghosh[2!], Shrestha Das[1!], Aditi Thakare[1!], Siddharth Singh[1], Apoorva Ganesh[1], Harsh Mahawar[1], Aadhya Jaisimha[1], Mohanapriya Krishna[1], Aritra Chattopadhyay[1], Rishima Borah[1], Vikrant Singh[1], Soumya M[2], Naveen Kumar[2], Sampath Kumar[2], Sunita Swain[2,*], and Suresh Subramani[3]

[1]Institute of Bioinformatics and Applied Biotechnology, Biotech Park, Electronic City Phase I, Bengaluru 560100, India

[2]Tata Institute for Genetics and Society (TIGS), Center at inStem, Bellary Road, GKVK Campus, Bengaluru 560065, India

[3]TIGS, University of California San Diego, La Jolla, CA 92093, USA

[!] these authors contributed equally to this paper

* sunita.swain@tigs.res.in and ssubha@ibab.ac.in (Corresponding Authors)

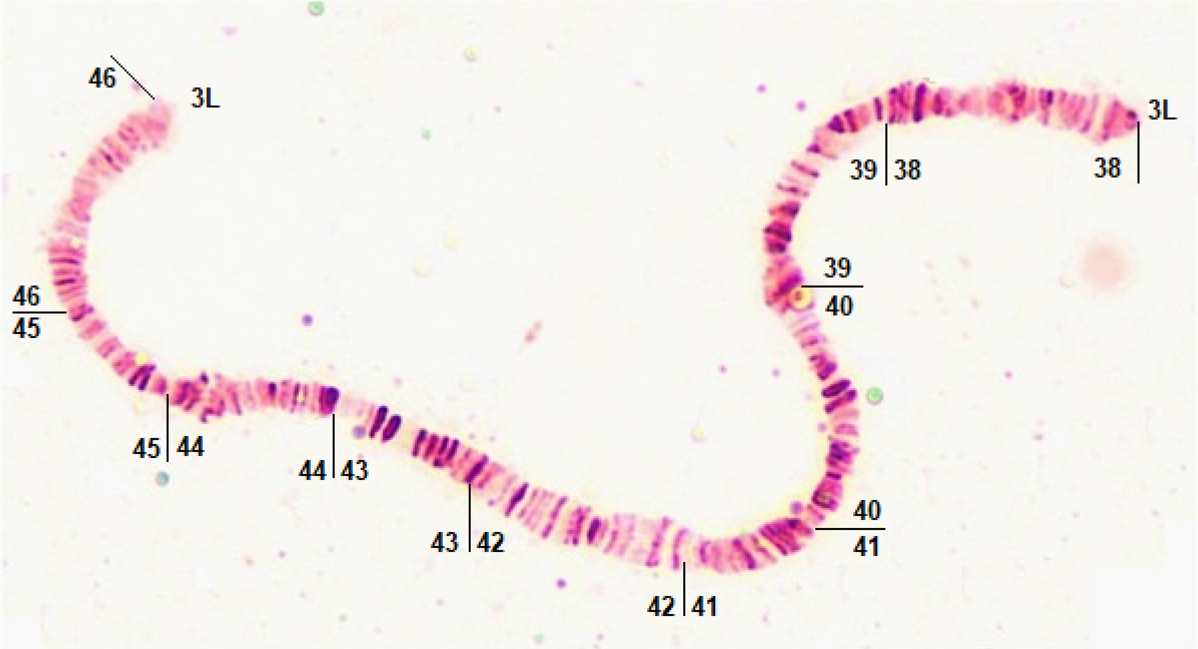


*Supplementary figure 1 : Photomap showing the standard form of the 3Li region in the IndInt strain, supporting 52% homozygous for the standard form of 3Li.*

**78 bp :**

```
Database: IndInt_chr2R.fasta
           1 sequences; 55,427,671 total letters



Query= IndCh_Astel_HiC_Chr2R

Length=55103326
                                                                      Score     E
Sequences producing significant alignments:                          (Bits)  Value

chr2R                                                                 331     1e-90


>chr2R
Length=55427671

 Score = 331 bits (179),  Expect = 1e-90
 Identities = 179/179 (100%), Gaps = 0/179 (0%)
 Strand=Plus/Minus

Query  38082990  GGCAGAAGTGAGTGAATGACTTAAATCTTTGAAATGAATTAGATGATTTAAATCATCACG  38083049
                 ||||||||||||||||||||||||||||||||||||||||||||||||||||||||||||
Sbjct  17244809  GGCAGAAGTGAGTGAATGACTTAAATCTTTGAAATGAATTAGATGATTTAAATCATCACG  17244750

Query  38083050  CAGCGTTATTTCAGCTCACTTTTGATTTAATTCGCCATGCCGATTAACCACTCACTCACT  38083109
                 ||||||||||||||||||||||||||||||||||||||||||||||||||||||||||||
Sbjct  17244749  CAGCGTTATTTCAGCTCACTTTTGATTTAATTCGCCATGCCGATTAACCACTCACTCACT  17244690

Query  38083110  ACTTTTTAACTCACTCACTATGAATGGCTCTCTCGTGAATTAAACGAGTGAGTTAAAGG  38083168
                 |||||||||||||||||||||||||||||||||||||||||||||||||||||||||||
Sbjct  17244689  ACTTTTTAACTCACTCACTATGAATGGCTCTCTCGTGAATTAAACGAGTGAGTTAAAGG  17244631
```

## 78 bp Reverse Complement :

```
Database: indint_rev_chr2R.fasta
           1 sequences; 55,427,671 total letters


Query= IndCh_Astel_HiC_Chr2R

Length=55103326
                                                                      Score        E
Sequences producing significant alignments:                          (Bits)     Value

chr2R                                                                 331        1e-90


>chr2R
Length=55427671

 Score = 331 bits (179),  Expect = 1e-90
 Identities = 179/179 (100%), Gaps = 0/179 (0%)
 Strand=Plus/Plus

Query  38082990  GGCAGAAGTGAGTGAATGACTTAAATCTTTGAAATGAATTAGATGATTTAAATCATCACG  38083049
                 ||||||||||||||||||||||||||||||||||||||||||||||||||||||||||||
Sbjct  38182863  GGCAGAAGTGAGTGAATGACTTAAATCTTTGAAATGAATTAGATGATTTAAATCATCACG  38182922

Query  38083050  CAGCGTTATTTCAGCTCACTTTTGATTTAATTCGCCATGCCGATTAACCACTCACTCACT  38083109
                 ||||||||||||||||||||||||||||||||||||||||||||||||||||||||||||
Sbjct  38182923  CAGCGTTATTTCAGCTCACTTTTGATTTAATTCGCCATGCCGATTAACCACTCACTCACT  38182982

Query  38083110  ACTTTTTAACTCACTCACTATGAATGGCTCTCTCGTGAATTAAACGAGTGAGTTAAAGG  38083168
                 |||||||||||||||||||||||||||||||||||||||||||||||||||||||||||
Sbjct  38182983  ACTTTTTAACTCACTCACTATGAATGGCTCTCTCGTGAATTAAACGAGTGAGTTAAAGG  38183041
```

## 8 bp :

```
Database: IndInt_chr2R.fasta
           1 sequences; 55,427,671 total letters


Query= IndCh_Astel_HiC_Chr2R

Length=55103326
                                                                      Score        E
Sequences producing significant alignments:                          (Bits)     Value

chr2R                                                                 202        6e-52


>chr2R
Length=55427671

 Score = 202 bits (109),  Expect = 6e-52
 Identities = 109/109 (100%), Gaps = 0/109 (0%)
 Strand=Plus/Minus

Query  21514292  ATTTAAGGTTGACCAATTCCTGAGAAACACGGGTAAGAATATAATCCAATCATATCTTTA  21514351
                 ||||||||||||||||||||||||||||||||||||||||||||||||||||||||||||
Sbjct  33742489  ATTTAAGGTTGACCAATTCCTGAGAAACACGGGTAAGAATATAATCCAATCATATCTTTA  33742430

Query  21514352  GCGAGCTGCAAATTAGTGATTTTCCTAGAACACTGGCAACACCGCCAAC  21514400
                 |||||||||||||||||||||||||||||||||||||||||||||||||
Sbjct  33742429  GCGAGCTGCAAATTAGTGATTTTCCTAGAACACTGGCAACACCGCCAAC  33742381
```

**8bp Reverse Complement :**

```
Database: indint_rev_chr2R.fasta
           1 sequences; 55,427,671 total letters


Query= IndCh_Astel_HiC_Chr2R

Length=55103326
                                                                      Score     E
Sequences producing significant alignments:                          (Bits)  Value

chr2R                                                                 202      6e-52


>chr2R
Length=55427671

 Score = 202 bits (109),  Expect = 6e-52
 Identities = 109/109 (100%), Gaps = 0/109 (0%)
 Strand=Plus/Plus

Query  21514292  ATTTAAGGTTGACCAATTCCTGAGAAACACGGGTAAGAATATAATCCAATCATATCTTTA  21514351
                 ||||||||||||||||||||||||||||||||||||||||||||||||||||||||||||
Sbjct  21685183  ATTTAAGGTTGACCAATTCCTGAGAAACACGGGTAAGAATATAATCCAATCATATCTTTA  21685242

Query  21514352  GCGAGCTGCAAATTAGTGATTTTCCTAGAACACTGGCAACACCGCCAAC  21514400
                 |||||||||||||||||||||||||||||||||||||||||||||||||
Sbjct  21685243  GCGAGCTGCAAATTAGTGATTTTCCTAGAACACTGGCAACACCGCCAAC  21685291
```

*Supplementary figure 2 : Pairwise alignment using BLASTN showing the validation of 2Rb breakpoints between the IndCh and IndInt strains.*

**g22432.t1**

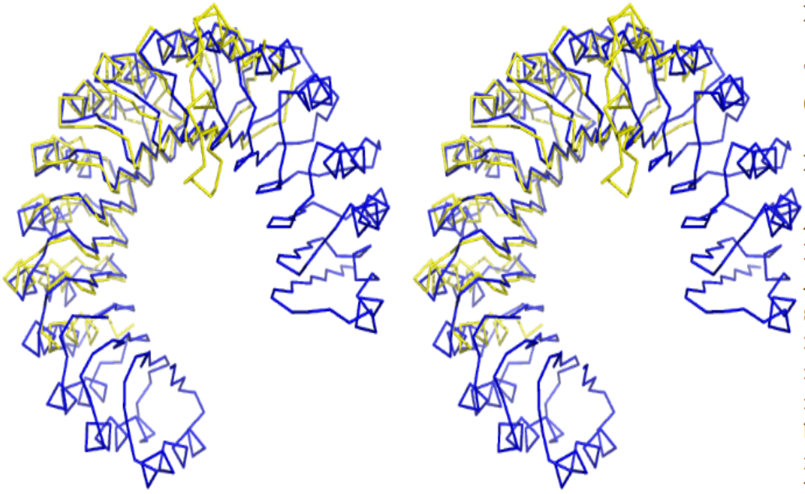

BLUE COLOR - DALI TEMPLATE

YELLOW COLOR-QUERY DOMAIN

DALI TEMPLATE ID - **1z7x-W**
DOMAIN LENGTH- **226-477 (251)**
DALI TEMPLATE LENGTH- **460**

The number of structurally equivalent residues (LALI) - **227 aa**

RMSD (DALI) - **2.3**

ANNOTATION - **LRR - Ribonuclease Inhibitor**
A leucine-rich repeat (LRR) is a protein structural motif that forms an α/β horseshoe fold. Leucine-rich repeats are frequently involved in the formation of protein–protein interactions.Leucine-rich repeat motifs have been identified in a large number of functionally unrelated proteins
Ribonuclease inhibitors (RI) or Ribonuclease/angiogenin inhibitor are a family of large proteins that bind to and inhibit ribonucleases.

*Supplementary figure 3a : Conserved domain structure of IndInt g22432*

**g22212.t1**

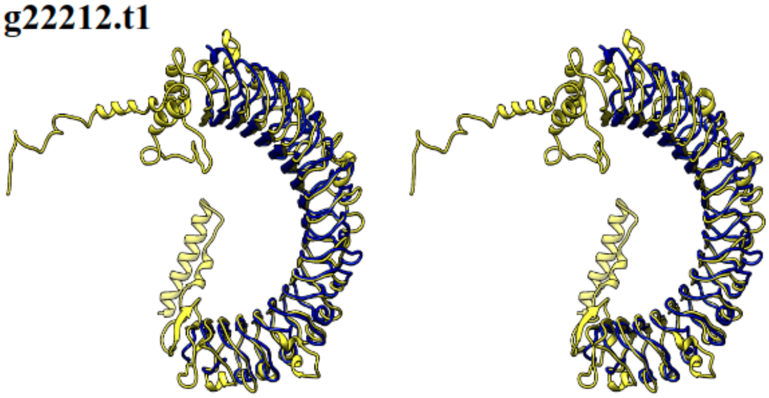

Function :

LEUCINE-RICH REPEAT-
CONTAINING G-PROTEIN COUPLED

DOMAIN 1 389-1021
(635 amino acid)

BLUE COLOR - DALI TEMPLATE

YELLOW COLOR-QUERY DOMAIN

DALI TEMPLATE ID - **4li1-B**

DOMAIN LENGTH- **635 AA**

LALI - **418 AA**

DALI TEMPLATE LENGTH- **425**

RMSD (DALI) - **2.6**

**g22212.t1**

DOMAIN 2 → 435-932
(497 amino acid)

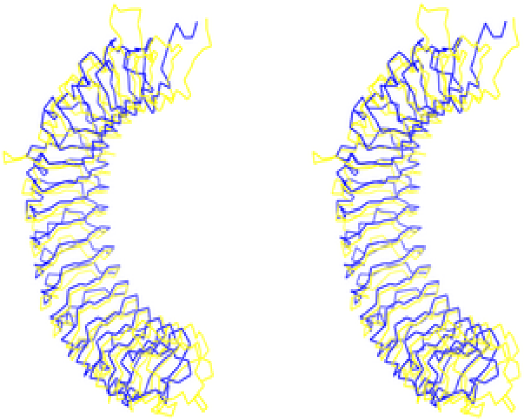

BLUE COLOR - DALI TEMPLATE

YELLOW COLOR-QUERY DOMAIN

DALI TEMPLATE ID - **4li1-B**

DOMAIN LENGTH- **497 AA**

LALI - **414 AA**

DALI TEMPLATE LENGTH- **425**

RMSD (DALI) - **2.5**

Function :

LEUCINE-RICH REPEAT-CONTAINING G-PROTEIN COUPLED

*Supplementary figure 3b : Conserved domain structure of IndInt g22212*

## g22118.t1

BLUE COLOR - DALI TEMPLATE

YELLOW COLOR-QUERY DOMAIN

DALI TEMPLATE ID - **1JB4-B**
DOMAIN LENGTH- 181-347 (**167 AA)**
DALI TEMPLATE LENGTH-**123 AA**

The number of structurally equivalent residues (LALI) - **120 AA**

RMSD (DALI) - **2.2**

ANNOTATION - **NTF2**
[**NTF2** is a cytosolic protein responsible for nuclear import of Ran, a small Ras-like GTPase involved in a number of critical cellular processes, including cell cycle regulation, chromatin organization during mitosis, reformation of the nuclear envelope following mitosis, and controlling the directionality of nucleocytoplasmic transport]

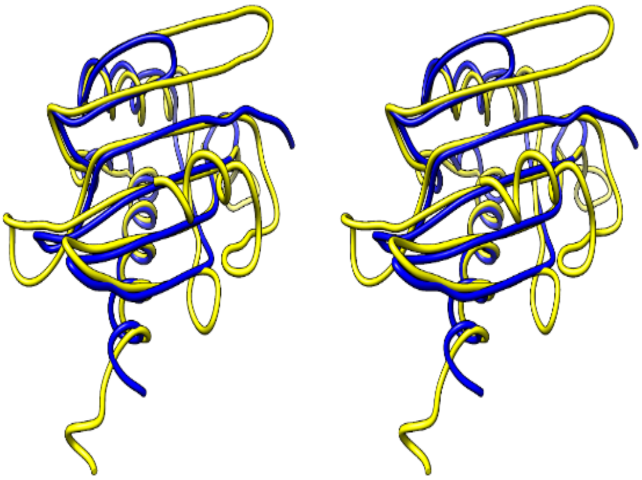

*Supplementary figure 3c : Conserved domain structure of IndInt g22118*

## g22089.t1

BLUE COLOR - DALI TEMPLATE

YELLOW COLOR-QUERY DOMAIN

DALI TEMPLATE ID - **5MBX-A**
DOMAIN LENGTH- 1-508 **(508 AA)**
DALI TEMPLATE LENGTH- **472 AA**

The number of structurally equivalent residues(LALI)- **433 AA**

RMSD (DALI) - **2.4**

ANNOTATION-
**PEROXISOMAL N1-(ACETYL)-SPERMINE/SPERMIDINE OXIDASE**

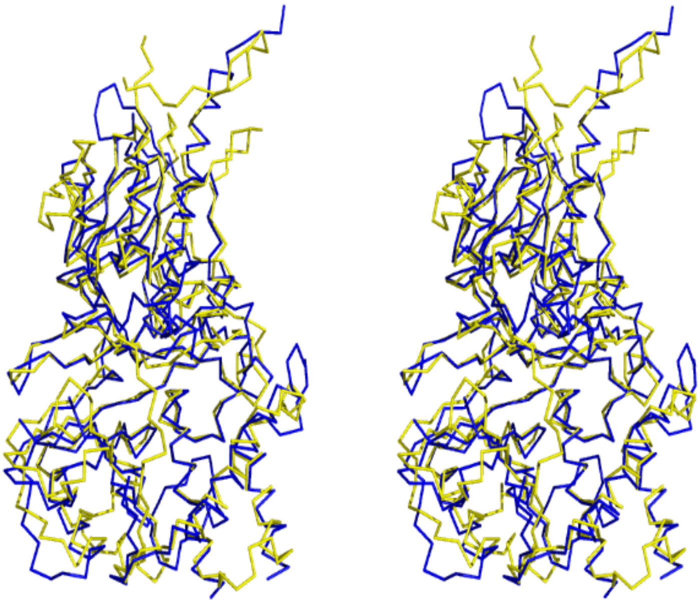

*Supplementary figure 3d : Conserved domain structure of IndInt g22089*

**g22220.t1**
**(Domain 3)**

BLUE COLOR - DALI TEMPLATE

YELLOW COLOR-QUERY DOMAIN

DALI TEMPLATE ID - **4J19-B**
DOMAIN LENGTH- 975-1042(**68 AA)**
DALI TEMPLATE LENGTH- **76 AA**

The number of structurally equivalent residues(LALI) - **65 AA**
RMSD (DALI) - **2.5**

ANNOTATION - **HOMEOBOX-CONTAINING PROTEIN 1**
[Binding to double-stranded telomere-associated DNA]

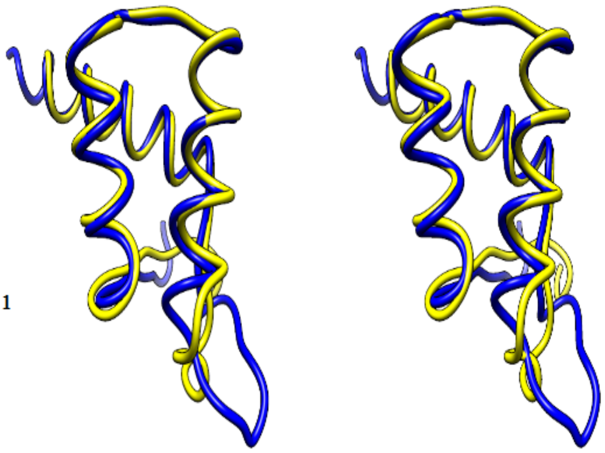

*Supplementary figure 3e : Conserved domain structure of IndInt g22220*

## g22349.t1

DOMAIN →153-405
(252 AMINO ACIDS)

BLUE COLOUR →DALI TEMPLATE

YELLOW COLOUR → QUERY DOMAIN

DALI TEMPLATE ID - **3I0P**

DOMAIN LENGTH- **252 AA**

LALI - **253 AA**

DALI TEMPLATE LENGTH- **361 AA**

RMSD (DALI) - **1.7**

FUNCTION - MALATE DEHYDROGENASE

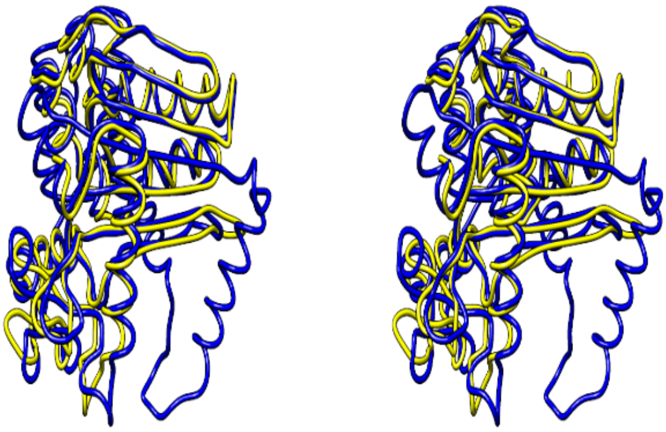

*Supplementary figure 3f : Conserved domain structure of IndInt g22349*

## g23051.t1

DOMAIN →189-468
(279 AMINO ACIDS)

BLUE COLOUR →DALI TEMPLATE

YELLOW COLOUR → QUERY DOMAIN

DALI TEMPLATE ID - **3SZ4**

DOMAIN LENGTH- **279 AA**

LALI - **180 AA**

DALI TEMPLATE LENGTH- **195 AA**

RMSD (DALI) - **2.6**

FUNCTION - EXONUCLEASE

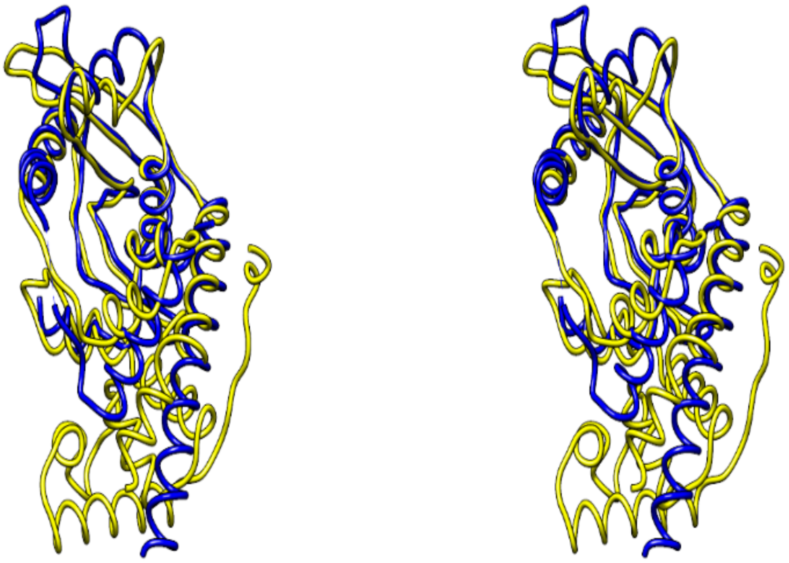

*Supplementary figure 3g : Conserved domain structure of IndInt g23051*

## g22565.t1

BLUE COLOR - DALI TEMPLATE

YELLOW COLOR-QUERY DOMAIN

DALI TEMPLATE NAME - **TUMOR NECROSIS FACTOR-INDUCIBLE GENE 6 PROTEIN**

DALI TEMPLATE ID - **2wno-A**
DOMAIN LENGTH - **128 aa**
DALI TEMPLATE LENGTH- **119 aa**

LALI - **110**

RMSD (DALI) - **2.0**

Percentage Coverage - **92.4%**
Sequence identity - **20%**

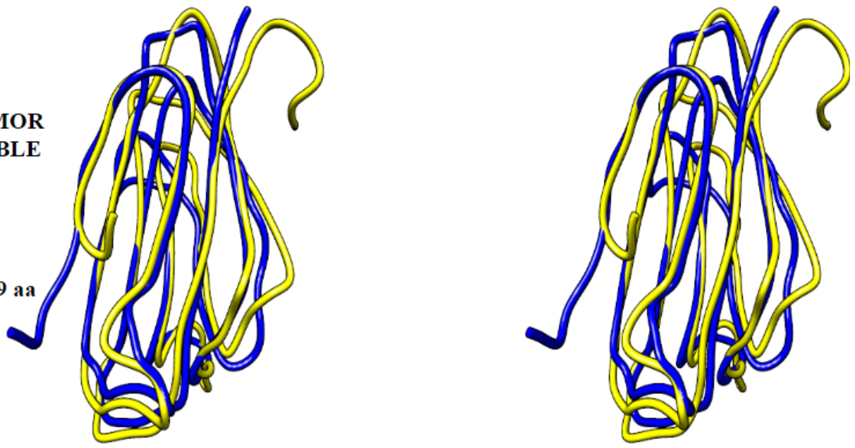

**g22565.t1**

BLUE COLOR - DALI TEMPLATE

YELLOW COLOR-QUERY DOMAIN
DALI TEMPLATE NAME - **COMPLEMENT C1R SUBCOMPONENT**

DALI TEMPLATE ID - 6f1d-A
DOMAIN LENGTH - 158
DALI TEMPLATE LENGTH- 117 aa

LALI - 112

RMSD (DALI) - **2.5**

Percentage Coverage ~ 71%
Sequence identity - 24%

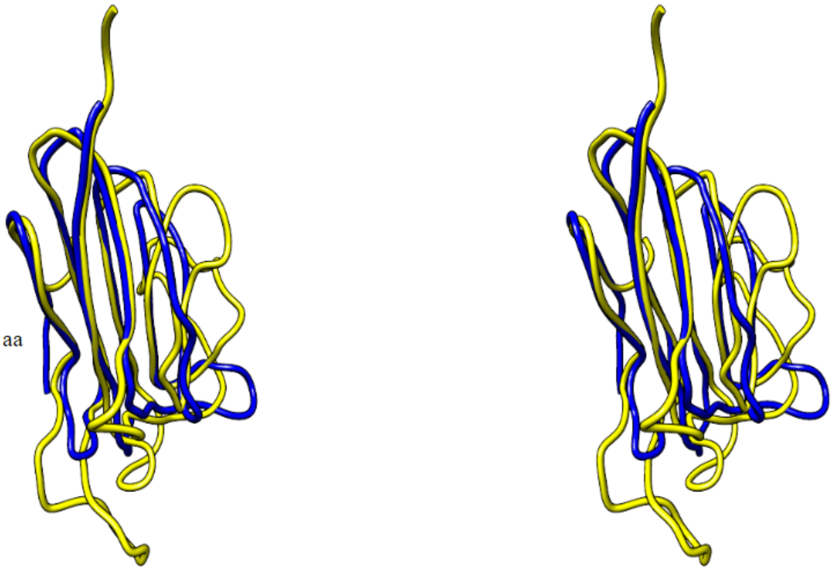

*Supplementary figure 3h : Conserved domain structure of IndInt g22565*

**g21982.t1**

BLUE COLOR - DALI TEMPLATE

YELLOW COLOR-QUERY DOMAIN
DALI TEMPLATE NAME - **UBIQUITIN**
DALI TEMPLATE ID - **2j7q-A**
DOMAIN LENGTH - 248
DALI TEMPLATE LENGTH- **232**
LALI - **177**
RMSD (DALI) - **3.1**

Percentage Coverage ~ 71.4%
Sequence identity - 24%

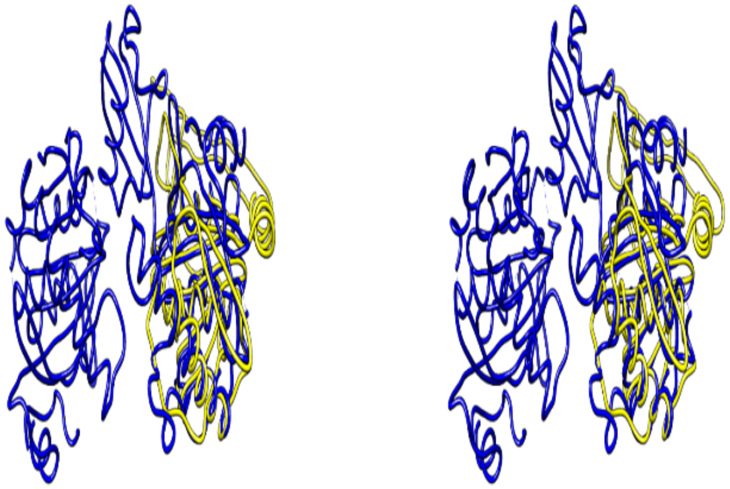

*Supplementary figure 3i : Conserved domain structure of IndInt g21982*

**g22147.t1**

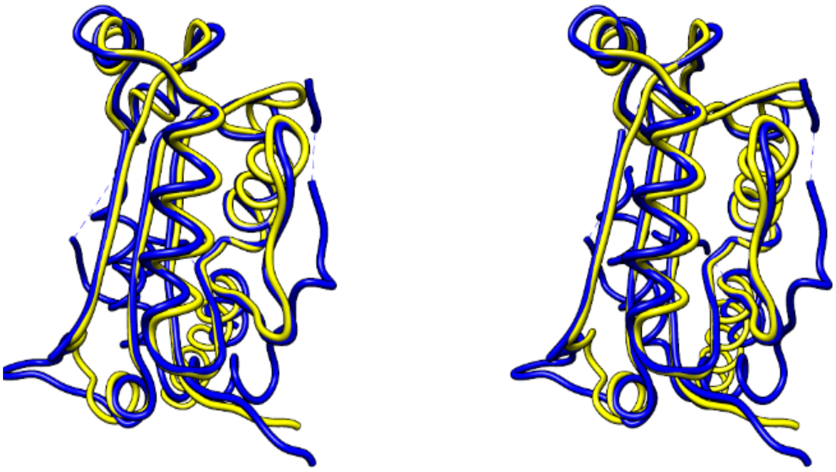

BLUE COLOR - DALI TEMPLATE

YELLOW COLOR-QUERY DOMAIN

DALI TEMPLATE ID - **1y97-B**
DOMAIN LENGTH- **1-156 (156)**
DALI TEMPLATE LENGTH- **211**

The number of structurally equivalent residues (LALI) - **145 aa**

RMSD (DALI) - **1.4**

ANNOTATION - **TREX2**
Exonuclease with a preference for double-stranded DNA with mismatched 3' termini. May play a role in DNA repair. Exonucleolytic cleavage in the 3'- to 5'-direction to yield nucleoside 5'-phosphates.

*Supplementary figure 3j : Conserved domain structure of IndInt g22147*

**g22088.t1**

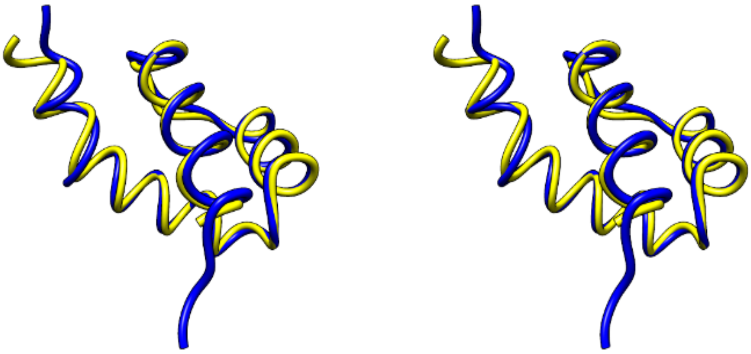

BLUE COLOR - DALI TEMPLATE

YELLOW COLOR-QUERY DOMAIN

DALI TEMPLATE ID - **6FQQ-B**
DOMAIN LENGTH- **240-297 (57)**
DALI TEMPLATE LENGTH- **61**

The number of structurally equivalent residues (LALI) - **57 aa**

RMSD (DALI) - **1.4**

ANNOTATION-**HOMEOBOX PROTEIN TGIF1**
A member of the three-amino acid loop extension (TALE) superclass of atypical homeodomains. TALE homeobox proteins are highly conserved transcription regulators. Both TGIF1 and TGIF2 act as transcription factors repressing TGF-β signalling.

*Supplementary figure 3k : Conserved domain structure of IndInt g22088*

**g22941.t1**

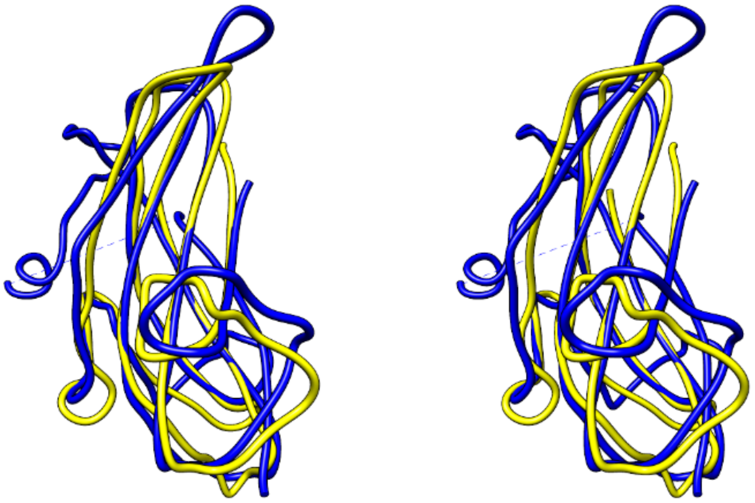

BLUE COLOR - DALI TEMPLATE

YELLOW COLOR-QUERY DOMAIN

DALI TEMPLATE ID - **4ajv-A**
DOMAIN LENGTH- **404-517 (113)**
DALI TEMPLATE LENGTH- **152**

The number of structurally equivalent residues (LALI) - **106 aa**

RMSD (DALI) - **2.1**

ANNOTATION - **TRANSFORMING GROWTH FACTOR BETA RECEPTOR TYPE 3**
Transforming growth factor beta (TGFβ) receptors are single pass serine/threonine kinase receptors that belong to TGFβ receptor family. TGFβ is a growth factor and cytokine involved in paracrine signalling and can be found in many different tissue types.

*Supplementary figure 3l : Conserved domain structure of IndInt g22941*

**g22536.t1**

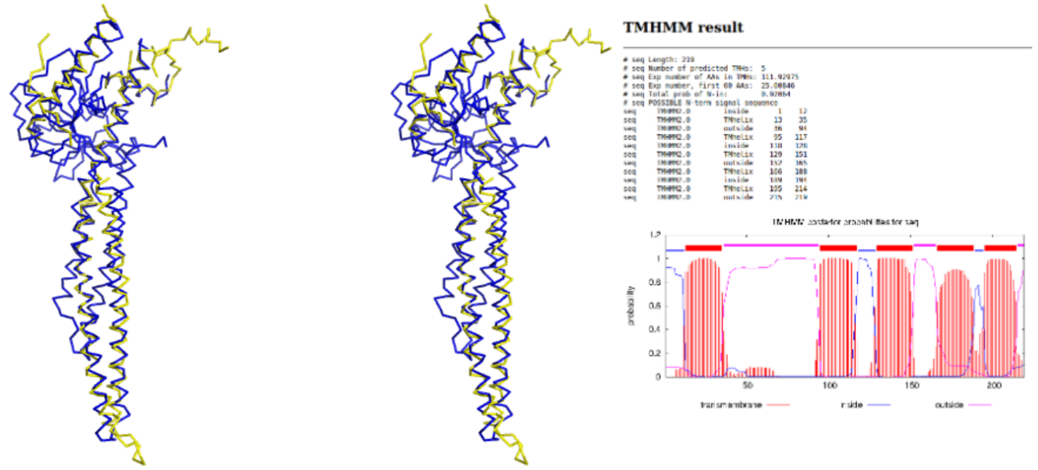


BLUE COLOR - DALI TEMPLATE

YELLOW COLOR-QUERY DOMAIN

DALI TEMPLATE ID - **6jfm-A**
DOMAIN LENGTH- **1-219 (219)**
DALI TEMPLATE LENGTH- **422**

The number of structurally equivalent residues (LALI) - **121**

RMSD (DALI) - **3.1**

ANNOTATION - **Mitofusin**
MFN2, an outer mitochondrial membrane GTPase, is critical for mitochondrial fusion, which in turn affects mitochondrial dynamics, distribution, quality control, and function.MFN2 modulates ER–mitochondria tethering.

*Supplementary figure 3m : Conserved domain structure of IndInt g22536*

**G22843**

BLUE COLOR - DALI TEMPLATE
YELLOW COLOR-QUERY DOMAIN

DALI TEMPLATE ID - **6FP9**
DOMAIN LENGTH- **209 AA**
DALI TEMPLATE LENGTH-**171 AA**
The number of structurally equivalent residues (LALI) - **168 AA**
RMSD (Chimera) - **1.897**
ANNOTATION - DARPin

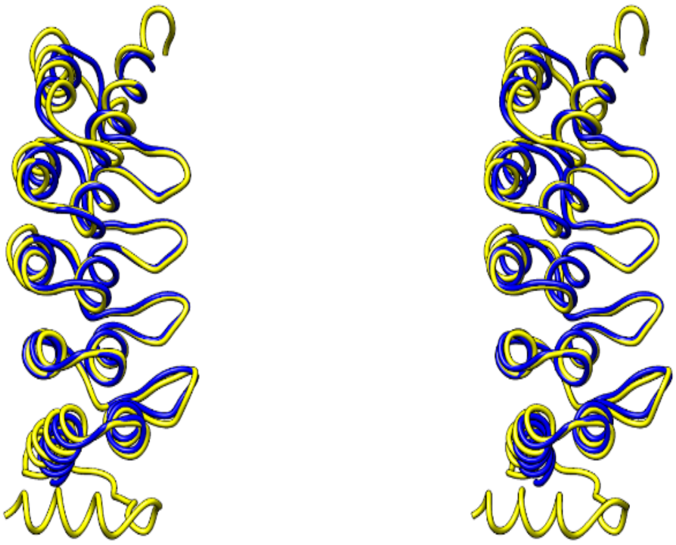

*Supplementary figure 3n : Conserved domain structure of IndInt g22843*

**G22150**

BLUE COLOR - DALI TEMPLATE
YELLOW COLOR-QUERY DOMAIN

**DALI TEMPLATE ID - 5WU1**
**DOMAIN LENGTH- 727 AA**
**DALI TEMPLATE LENGTH-493 AA**
The number of structurally equivalent residues (**LALI**) - **353 AA**
**RMSD** (Chimera) - **2.5**

**ANNOTATION -**

SPECKLE TARGETED PIP5K1A-REGULATED POLY(A) POLYMERASE.
Poly(A) polymerase that creates the 3'-poly(A) tail of specific pre-mRNAs

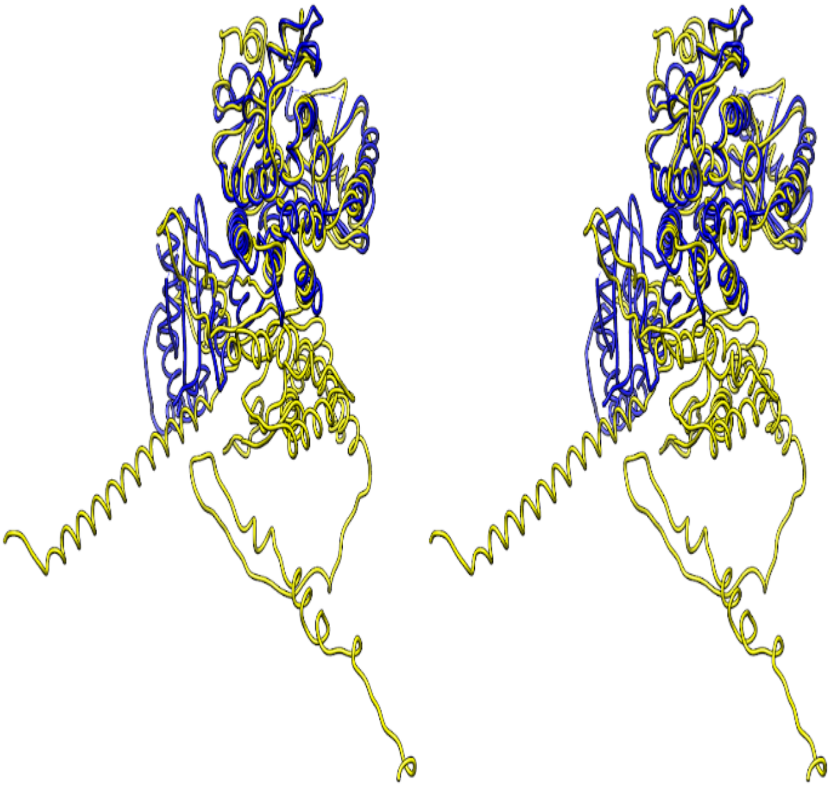

*Supplementary figure 3o : Conserved domain structure of IndInt g22150*

**DOMAINS WHICH DID NOT SUPERIMPOSE WELL BUT HAVE A FUNCTIONAL ANNOTATION IN DALI**

GENE ID - **g22118.t1**
DOMAIN LENGTH - 1-109(**109 AA**)

FIRST HIT DALI TEMPLATE PDB ID - **7K3R-A**
DALI TEMPLATE LENGTH- **95 AA**

The number of structurally equivalent residues(LALI) - **80 AA**

RMSD (DALI) - **3.5**

ANNOTATION - **INTERFERON INDUCIBLE PROTEIN AIM2**

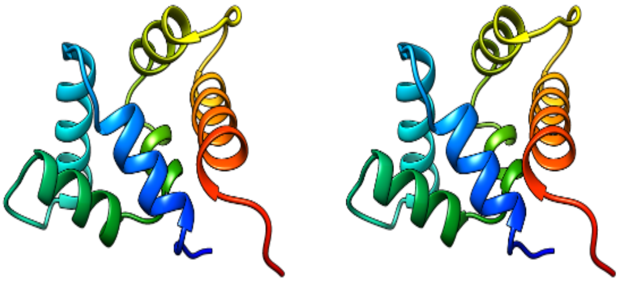

*Supplementary figure 3p : DALI functional annotation of IndInt g22118*

**G22810.t1 (not superimposed well with template)**

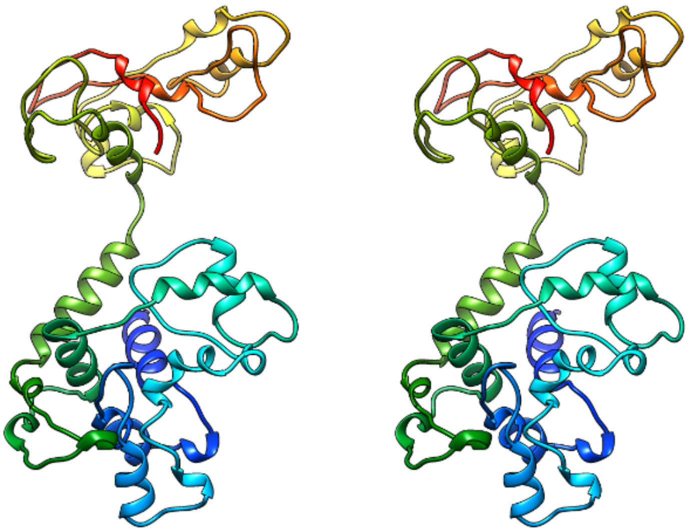

DOMAIN 1
246-561
(315 amino acid)

DALI TEMPLATE ID - **7lw7-A (only one hit )**

DOMAIN LENGTH- **315 AA**

LALI - **80 AA**

DALI TEMPLATE LENGTH- **277**

RMSD (DALI) - **5.7**

Function :
EXONUCLEASE V
The Exonuclease V enzyme is an ATP-dependent, double-strand DNA exonuclease.

*Supplementary figure 3q : DALI functional annotation of IndInt g22810*

**g22301.t1 (not superimposed well with template)**

DOMAIN 1
21-306
(285 amino acid)

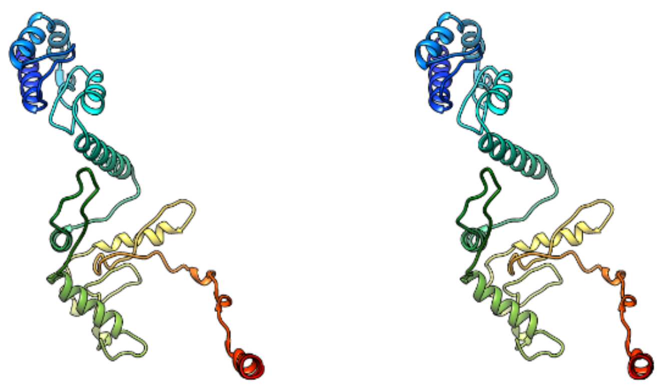

DALI TEMPLATE ID - **2v71-A**
DOMAIN LENGTH- **285 AA**

LALI - **156 AA**

DALI TEMPLATE LENGTH- **160**

RMSD (DALI) - **5.2**

Function :
NUCLEAR DISTRIBUTION PROTEIN NUDE-LIKE 1
It plays a role in multiple processes including cytoskeletal organization, cell signaling and neuron migration, outgrowth and maintenance

*Supplementary figure 3r : DALI functional annotation of IndInt g22301*

**g23001 (not superimposed properly)**

DOMAIN
115-395
(280 amino acid)

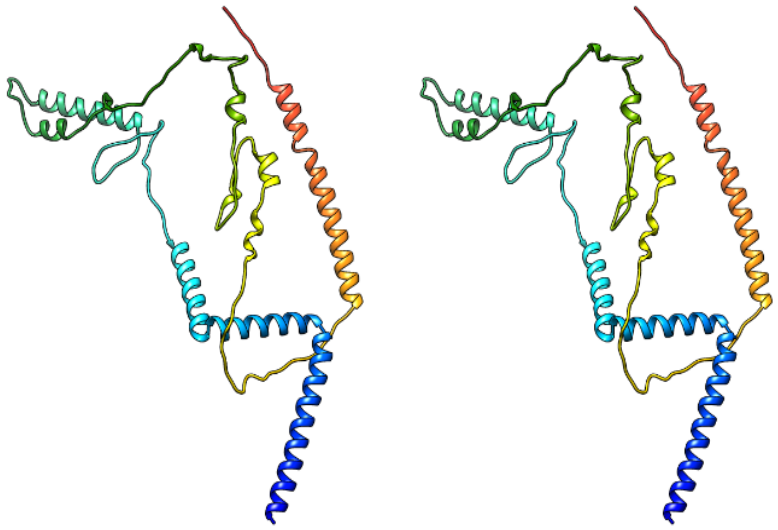

DALI TEMPLATE ID - **1xpj-D**
DOMAIN LENGTH- **280 AA**

LALI - **106 AA**

DALI TEMPLATE LENGTH- **123**

RMSD (DALI) - 2.6

The Hit template is a HYPOTHETICAL PROTEIN;

*Supplementary figure 3s : DALI functional annotation of IndInt g23001*

## MODELS WHICH DID NOT FOLD WELL-

**MODELS WHICH DID NOT FOLD WELL**

**g22846.t1(length - 701 AA)**

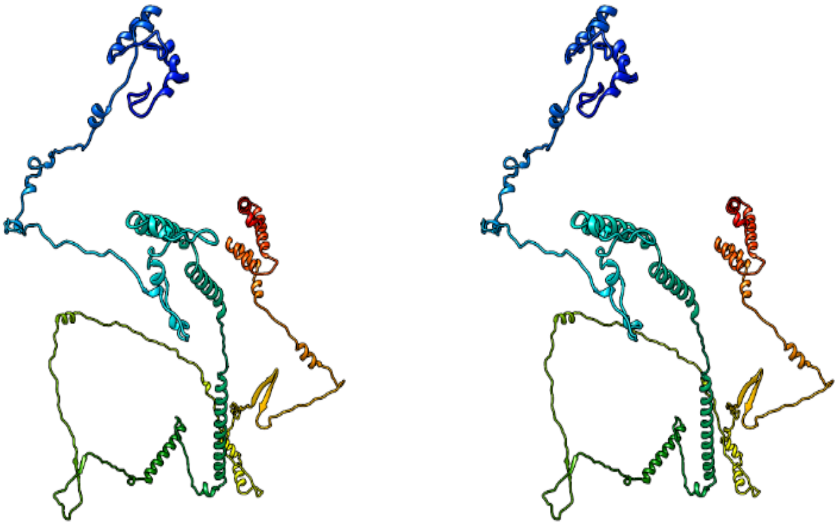

*Supplementary figure 3t : Alpha fold predicted structure of IndInt g22846*

**g22224.t1 (LENGTH - 343 AA)**

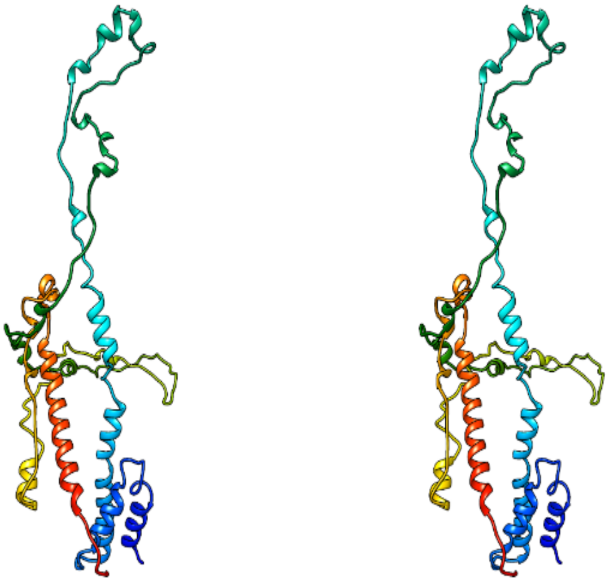

*Supplementary figure 3u : Alpha fold predicted structure of IndInt g22224*

**g22323 450 amino acids - very high error plot**

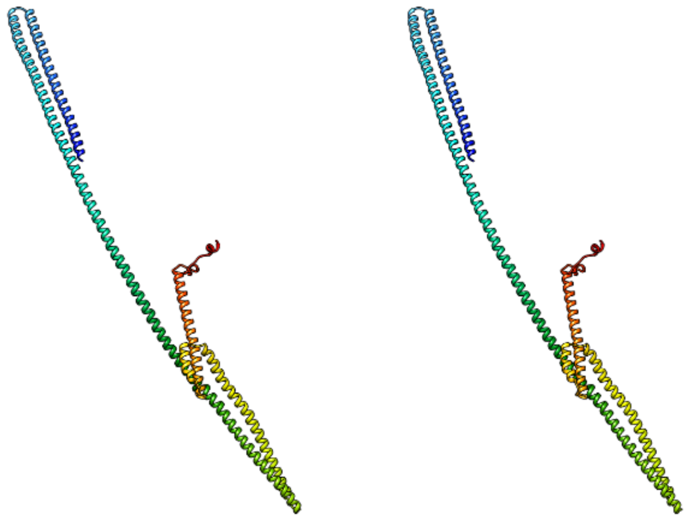

*Supplementary figure 3v : Alpha fold predicted structure of IndInt g22323*

**g21978.t1**

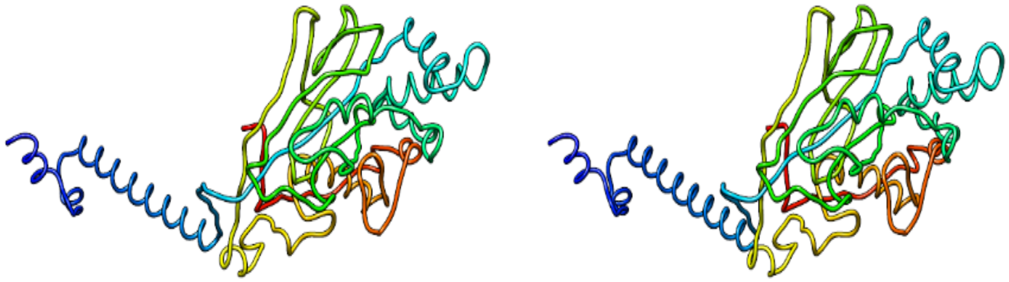

*Supplementary figure 3w : Alpha fold predicted structure of IndInt g21978*

**g22197.t1**

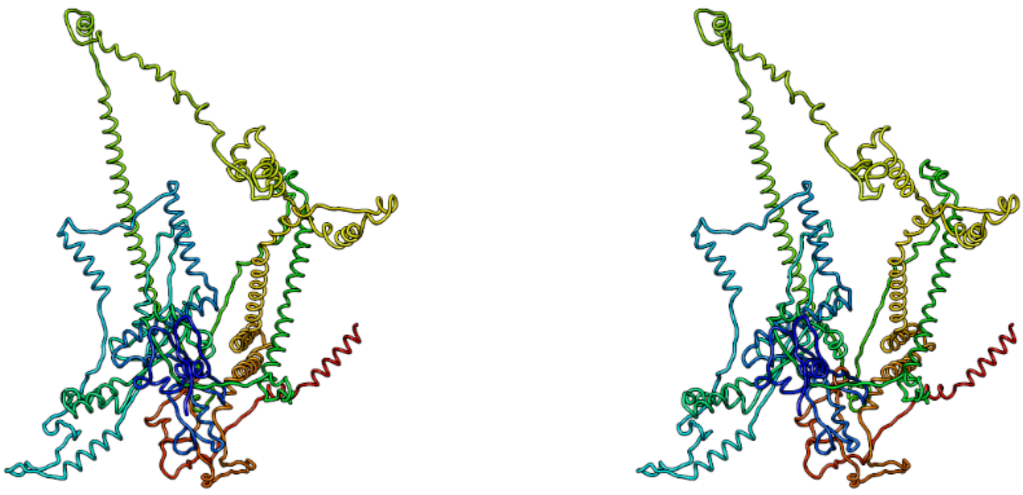

*Supplementary figure 3x : Alpha fold predicted structure of IndInt g22197*

**g22416.t1**

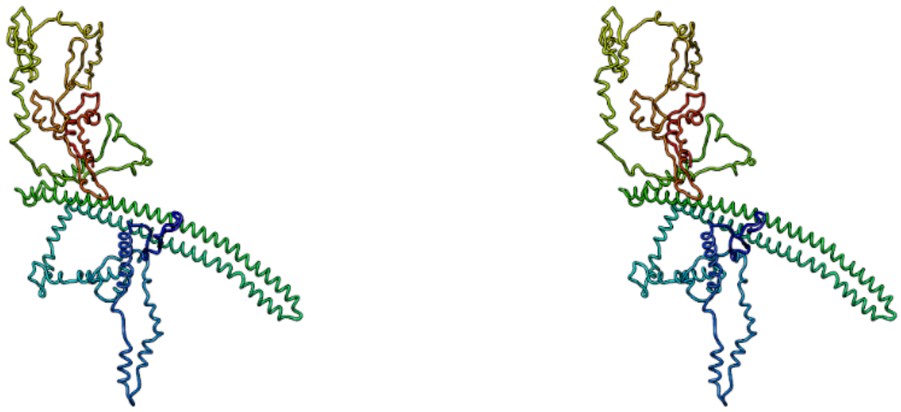

*Supplementary figure 3y : Alpha fold predicted structure of IndInt g22416*

**g22689.t1**

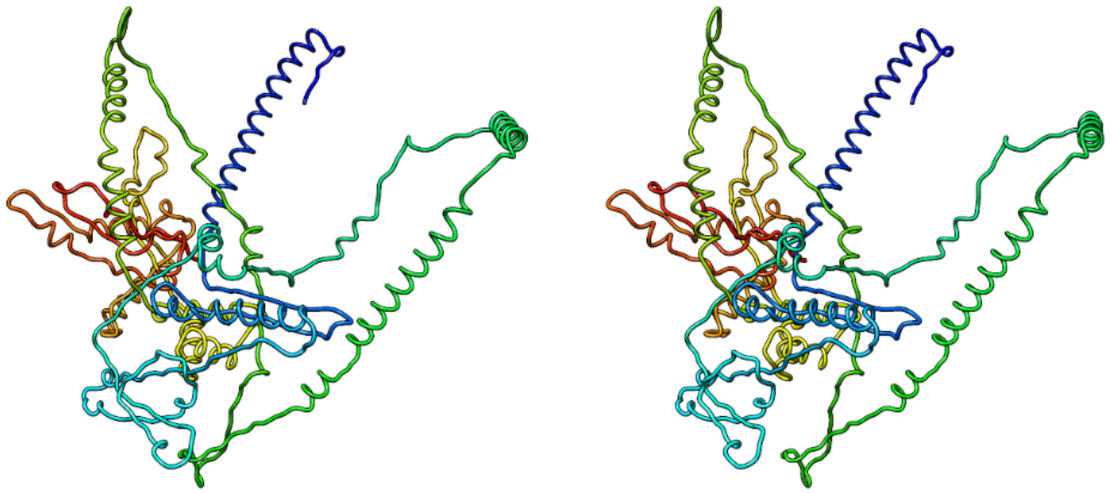

*Supplementary figure 3z : Alpha fold predicted structure of IndInt g22689*

**g21989.t1**

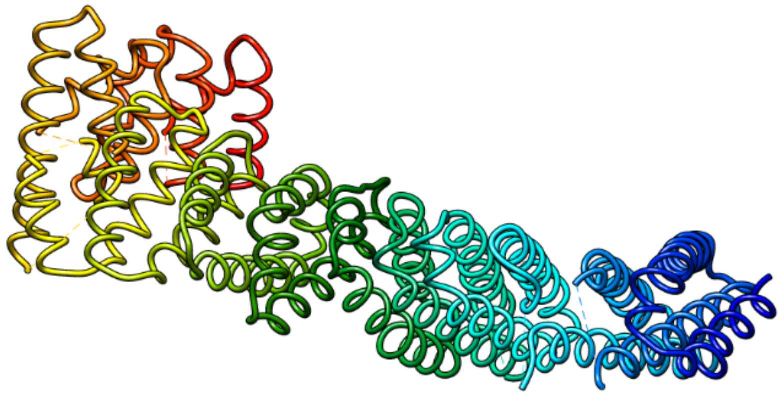

*Supplementary figure 3a1 : Alpha fold predicted structure of IndInt g21989*

**g22713.t1**

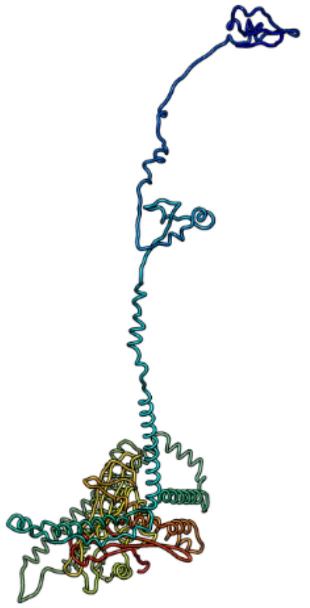

*Supplementary figure 3a2 : Alpha fold predicted structure of IndInt g22713*

**g23157.t1**

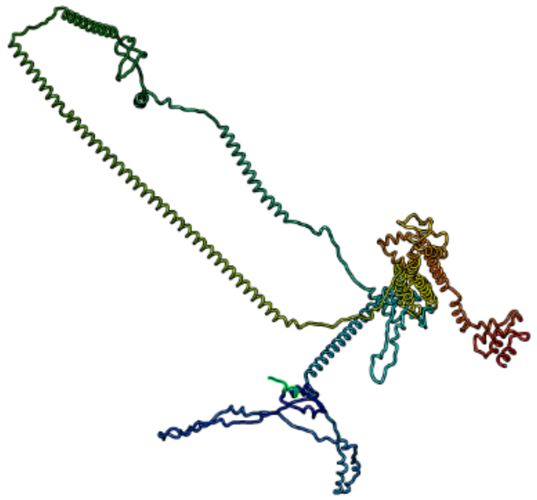

*Supplementary figure 3a3 : Alpha fold predicted structure of IndInt g23157*

**g22302.t1**

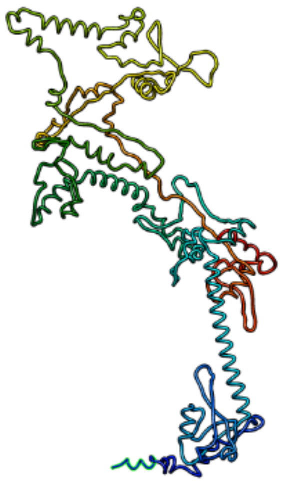

*Supplementary figure 3a4 : Alpha fold predicted structure of IndInt g22302*

**g22313.t1**

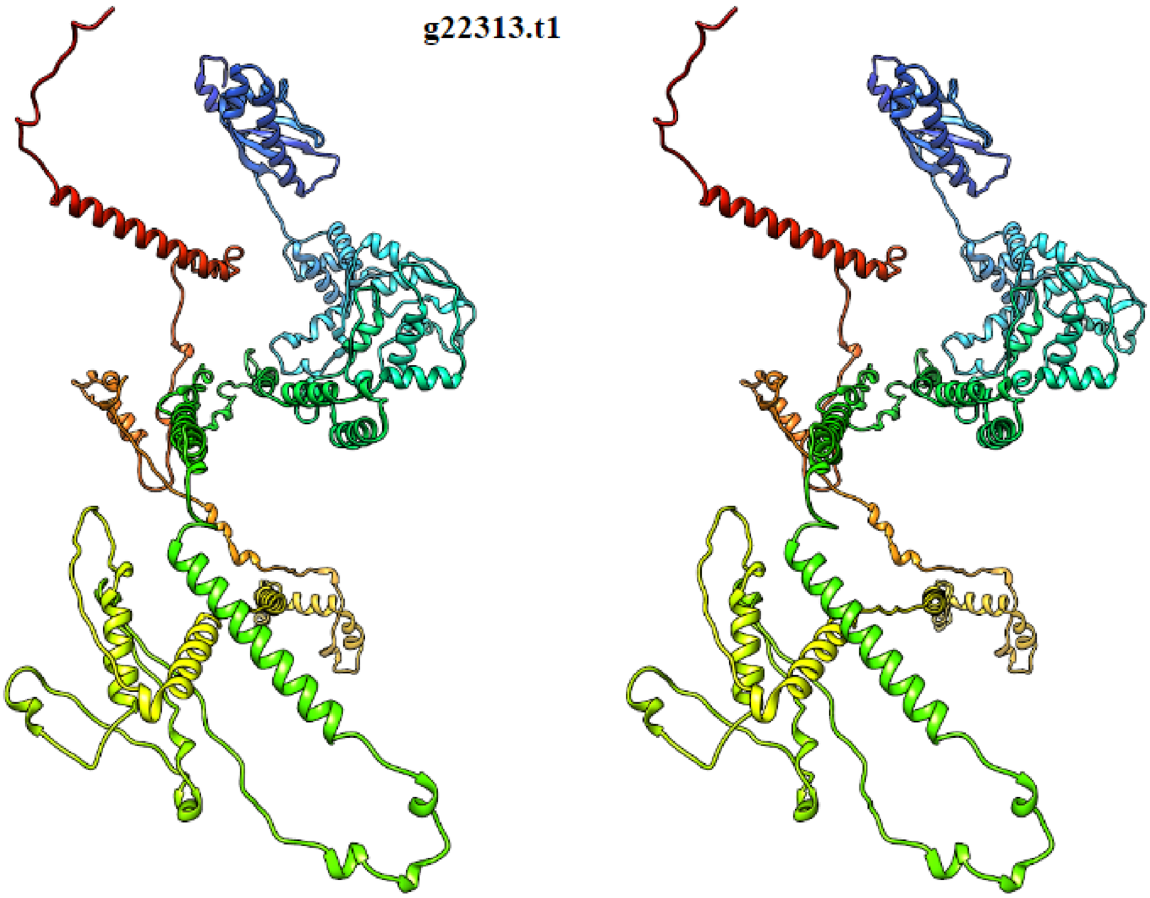

*Supplementary figure 3a5 : Alpha fold predicted structure of IndInt g22313*

CLUSTAL O(1.2.4) multiple sequence alignment

```
AnColuzzii_XP040229266          -----------------------------------------------MSDTNLLQLSAAW      13
AnArabiensis_XP040157989        -----------------------------------------------MSDTNLLQLSAAW      13
AnGambiae_AGAP005805            -----------------------------------------------MSDTNLLQLSAAW      13
IndInt_g22432                   -----------------------------------------------MSDTNLLHLSALW      13
AnStephensi_XP035917226         MFRISHATDSFDPAADVRIIEALKLLHQVQTEPSKGIAQPISCERIKMSDTNLLHLSALW      60
                                                                               *******:*** *

AnColuzzii_XP040229266          TQLPRLMVPFDRQLSNALRTWAPSWQTQELASETTRKLFVELVTFGGVNQCRRFDALARP      73
AnArabiensis_XP040157989        RQLPRLMVPFDRQLSNALRTWAPSWQTQQLASETTRKLFVELVTFGGVNQCRRFDALARP      73
AnGambiae_AGAP005805            RQLPRLMVPFDRQLSNALRTWAPSWQTQQLASETTRKLFVELVTFGGVNQCRRFDALARP      73
IndInt_g22432                   RQLPHLTSPLERELWNAFREYGPSWQTQQLASETQRELFGELVAFGGRRACRPFDAMVHV      73
AnStephensi_XP035917226         RQLPHLTSPLERELWNAFRAYGPSWQTQQLASETQRELFVELVAFGGRRTCRPFDAMVHV      120
                                 ***:*  *::*:* **:* :.******:***** *:** ***:*** . ** ***:.:

AnColuzzii_XP040229266          VINDHGEGVVPPRLSTIVVQFLVTRYDGGPEPTALSCCQVRDFGCLLSTELPLLKIVDLE      133
AnArabiensis_XP040157989        VINDHGEGVVPARLSTIVVQFLVARYDGGPEPAALSCCQVREFGCLLSTELPLLKIVDLE      133
AnGambiae_AGAP005805            VINDHGEGVVPPRLSTIVVQFLVTRYDGGPEPTALSCCQVREFGCLLSTELPLLKIVDLE      133
IndInt_g22432                   --TDDIVQDVPKRLSTLVLEFLVASFDGGPEPGPLNCCQVREFGSLLSTELPLIKVIELE      131
AnStephensi_XP035917226         --TDDIVQDVPKRLSTLVLEFLVVSFDGGPEPGPLSCCQVREFGSLLSTELPLIKVIELE      178
                                  .*.    ** ****:*::***. :******  *.*****:**.********:*:::**

AnColuzzii_XP040229266          SETYWRRVVWCYTDEPLSYYEHDYLTPHRFWKQHGVELKLARMIEQQDPMYWELEGLEDT      193
AnArabiensis_XP040157989        SETYWRRVVWCYADEPLSYYEHDYLTPRRFWKQHGIELKLARMIEQQDPVYWELEGLEDT      193
AnGambiae_AGAP005805            SETYWRRVVWCYADEPLSYYEHDHLTPRRFWKQHGVELKLARMIEQQDPVYWELEGLEDT      193
IndInt_g22432                   SETYWRRVVWCYSNDSLSYHELDYLSPRRFWKQQGIELKLARMIEQQDPQYWELEGLEET      191
AnStephensi_XP035917226         SETYWRRVVWCYSNDSLSYHELDYLSPRRFWKQQGIELKLARMIEQQDPHYWELEGLEET      238
                                ************::: ***:* *:*:*:*****:*:************* ********:*

AnColuzzii_XP040229266          IKRAASFVNNLVVQQLRPYAMVEPLEDYEAYTIRNAPADLCHHGSLAVLGHLVNLTSLSL      253
AnArabiensis_XP040157989        IKRAAPFVNNLVVQQLRPYAMVEPLEDYEAYTIRNAPADLCHHGSLAVLGHLVNLTSLSL      253
AnGambiae_AGAP005805            IKRAAPFVNNLVVQQLRPYAMVEPLEDYEAYTIRNAPADLCQHGSLAVLGHLVNLTSLSL      253
IndInt_g22432                   IKKAAPFVDTLYVEQLQPLPTVEPIEDYEEYHIRNAPDELCNHGSLAILGHLVNLTSLSL      251
AnStephensi_XP035917226         IKRAAPFVDTLYIEQLQPHPTVEPIEDYEEYHIRNAPDELCNHGSLAILGHLVNLTSLSL      298
                                **:** **:.* ::**:*   ***:**** * ***** :**:*****:************

AnColuzzii_XP040229266          VFGVKHWTKAYQTRYSQCSQTDIEQLGMGLQKLVKLKKFNISHTRLNASKLKALLESLTP      313
AnArabiensis_XP040157989        MFGVKHWTKAYQTRYSHCSQTDIEQLGMGLQKLVKLKKFNLSHTRLNASKLKPLLESLTP      313
AnGambiae_AGAP005805            VFGVKHWNKAYQTRYSHCSQTDIEQLGMGLQKLVKLKKFNLSHTRLNASKLKALLESLTP      313
IndInt_g22432                   VFGVKHWTKPYQNRFSSCSLADIENLGQGLQKLHRLKKFTLSHTQLNSAKLKILLEFLTP      311
AnStephensi_XP035917226         VFGVKHWIKPYQNRFSSCSLADIENLGQGLQKLHRLKKFTLSHTQLNSAKLKILLEFLTP      358
                                :****** * **.*:* ** :***:** ***** :****.:***:**::*** *** ***

AnColuzzii_XP040229266          LGLEAIRLTHCHLGEGCGGPLGRFLSRFGPTLKQLDLSNNRLDAPELDQLCPGLSIYQGT      373
AnArabiensis_XP040157989        LGLETIRLTHCHLGEGCGGPLGRFLSRFGPTLKQLDLSNNRLDAPELDQLCPGLSIYQGT      373
AnGambiae_AGAP005805            LGLETIRLTHCHLGEGCGGPLGRFLSRFGPTLKQLDLSNNRLDAPELDQLCPGLSIYQGK      373
IndInt_g22432                   LELETIRLTHCQLAAGCGAILGRFLSRFQPTLTQLDLSHNRLDAPELDQLCPGLSVYRGV      371
AnStephensi_XP035917226         LELETIRLTHCQLAAGCGAILGRFLSRFQPTLTHLDLSHNRLDAPELDQLCPGLSVYRGV      418
                                * **:******:*. ***. ******** ***.:****:****************:*:*
```

```
AnColuzzii_XP040229266       LDKLNLSYNPIGEAGVLILGGAIKGRAQLSELHFTGCPMGVEGSFRVIQLLSFHETLRKV     433
AnArabiensis_XP040157989     LDKLNLSYNPIGEAGVLILGGAIKGRAQLSELHFTGCPMGVEGSFRVIQLLSFHETLRKV     433
AnGambiae_AGAP005805         LDKLNLSYNPIGQAGVLILGGAIKGRAQLSELNFTGCPLGVEGSFRVIQLLSFHETLRKV     433
IndInt_g22432                VDRLDLSYNPIGEAGVLILGGAIKGRTQLSELNFTGCQMGVEGSFRL---LSFHVTLRKV     428
AnStephensi_XP035917226      VDRLDLSYNPIGEAGVLILGGAIKGRTQLSELNFTGCQMGVEGSFRVIQLLSFHVTLRKI     478
                             :*:*:*******:*************:*****:**** :*******:   **** ****:

AnColuzzii_XP040229266       SLNCVPISPEGGDKLVQVLLENNRIEEVQVRDCGLTERVRSKIGKILRKNASTRHQSRRS     493
AnArabiensis_XP040157989     SLNCVPISPEGGDKLVQVLLENNRIEEVQVRDCGLTERVRARIGKILRKNANARHRSRRS     493
AnGambiae_AGAP005805         SLNCVPISPEGGDKLVQVLLENNRIEEVHVRDCGLTERVRVRIGKILRKNANARHRSRRS     493
IndInt_g22432                SLNCVPISLEGGAKLVQVLQENIRIEDVQVRQCGLPDELLVKPHIIFANPDYRASSSGDT     488
AnStephensi_XP035917226      SLNCVPISLEGGAKLVQVLQENIRIEDVQVRQCGLPDELLVKVRKILRKNAKIRDQLR-S     537
                             ******** *** ****** ** ***:*:**:*** :.:  :   *: :          :

AnColuzzii_XP040229266       DGKVATPPMVRSPAT-------------------MFQLERQVFPVQS-------------     521
AnArabiensis_XP040157989     DGKVATPPMVRSPAS-------------------MFQLERQVFPVQS-------------     521
AnGambiae_AGAP005805         AGKVATPPMVRSPAS-------------------MFQLERQVFPVQS-------------     521
IndInt_g22432                ALLTAPKEIRHCPPVDRQAEPVPGRTIIHYANKSHFPFPVRILVKVLRPVAFSGPTRTKL     548
AnStephensi_XP035917226      VRMSSEQPMDKCHTSERSLGS-------------MFLLERQ---------VFSE------     569
                                 :   : :.                       * :  :

AnColuzzii_XP040229266       ------------------------------------------------------------     521
AnArabiensis_XP040157989     ------------------------------------------------------------     521
AnGambiae_AGAP005805         ------------------------------------------------------------     521
IndInt_g22432                CRVAPGPTFASASDSEQTLALWSECNTTRADRSTADRPIRSRSIGGLIVKWECLWHGPEH     608
AnStephensi_XP035917226      ------------------------------------------------------------     569

AnColuzzii_XP040229266       ------------------------------------------------------------     521
AnArabiensis_XP040157989     ------------------------------------------------------------     521
AnGambiae_AGAP005805         ------------------------------------------------------------     521
IndInt_g22432                KRMCACASVCVLCMWAPRFGRHFDGSMTLKTSTGNIYQTGYRLSGTSGRITSACFTRVHP     668
AnStephensi_XP035917226      ------------------------------------------------------------     569

AnColuzzii_XP040229266       ------------------------- 521
AnArabiensis_XP040157989     ------------------------- 521
AnGambiae_AGAP005805         ------------------------- 521
IndInt_g22432                GTICSGFMATGERHTHAHIPPTTRK 693
AnStephensi_XP035917226      ------------------------- 569
```

*Supplementary figure 4a : MSA for IndInt gene g22432.t1 across other Anopheles species shows high conserved homology.*

```
CLUSTAL O(1.2.4) multiple sequence alignment


AnColuzzii_XP040230463       ------------------------------------------------------------     0
AnArabiensis_XP040158968     ------------------------------------------------------------     0
AnGambiae_AGAP006645         ------------------------------------------------------------     0
AnStephensi_XP035917013      ------------------------------------------------------------     0
IndInt_g22212                MRGLTRSVDPTARLQDHETERTVVCAYGYIGIPDISFWQLVNAPLRESEMASEPYTVPDS     60


AnColuzzii_XP040230463       --------------------------------------------------MVSVHTMRCQ     10
AnArabiensis_XP040158968     --------------------------------------------------MVSVHTMRCQ     10
AnGambiae_AGAP006645         --------------------------------------------------------MRCQ     4
AnStephensi_XP035917013      --------------------------------------------------------MSRQ     4
IndInt_g22212                GCMAMHSRKSVEECRVQSRNDGGFSPARMQTPPWYNICSNLTVPLIFFTQQGSIHTMSRQ     120
                                                                                     *  *

AnColuzzii_XP040230463       YLLVLTVVLCALTVN---SLKPDLPKAKDPLFNKMLAESSNKGVDAQDPRYNKMLPDLEE     67
AnArabiensis_XP040158968     YLLVLTVVLCALTVN---SLKPDLPKAKDPLFNKMLAESSTKGVDVQDPRYNKMLPDLEE     67
AnGambiae_AGAP006645         YLLVLTVVLCALTVN---SLKPDLPKAKDPLFNKMLAESSTKGVDAQDPRYNKMLPDLEE     61
AnStephensi_XP035917013      YLLVLAVALCALAVHSSPALKPDLPKAQDPLYNKILAETTRSVMDAHDRHYNKMLPQPDE     64
IndInt_g22212                YLLVLAVALCALAVNSSPALKPDLPKAQDPLYNKILAETTRSVMDAHDRHYNKMLPQPDE     180
                             *****:*.****:*:   :********:***:**:***:: . :*.:* :******: :*

AnColuzzii_XP040230463       NLIDDDDEDDDEEEEDDV----APPTKKAADLNPMYALRGKQ-VKPEVSTVGSVAINKVK     122
AnArabiensis_XP040158968     NLIDDDDEDDDEEEEDDV----APPTKKAADLNPMYALRGKQ-VKPEVSTVGSVAINKVK     122
AnGambiae_AGAP006645         NLIDDDDEDDDEEEEDDV----APPTKKAADLNPMYALRGKQ-VKPEVSTVGSVAINKVK     116
AnStephensi_XP035917013      NLINSDISEDEDGDEDDAMANDDILPKKAADLNPMYAQRGKQPAKPEVSTVGSVAINKVK     124
IndInt_g22212                NLINSDISEDEDADDDEAMANDDILPKKAADLNPMYAQRGKQPAKPEVSTVGSVAINKVK     240
                             ***:.* .:*:: ::*:.        *********** **** .****************

AnColuzzii_XP040230463       INEDIDSYEEVLLKGNGKSV--------------KPSKSTTAKPVAKPATEDNYDEYDDD     168
AnArabiensis_XP040158968     INEDIDSYEEVLLKGNGKSV--------------KPSKSTTAKPVAKPATEDNYDEYDDD     168
AnGambiae_AGAP006645         INEDIDSYEEVLLKGNGKSVDIDDSYQVEDLSGEKPSKSTTAKPVAKPATEDNYDEYDDD     176
AnStephensi_XP035917013      INEDIDSYEEVLLKGNGKSV--------------KPSKPTTSKPAAKTEGED--DE-YDD     167
IndInt_g22212                INEDIDSYEEVLLKGNGKSV--------------KPSKPTTSKPAAKTEGED--DE-YDD     283
                             ********************              **** **:**.**   **  **  **

AnColuzzii_XP040230463       ESDEQIDFSGVDKVLAQPLKPQVPAEKSAKPAVPSTTAKPKAVTTGKQNVEILDQYDDDA     228
AnArabiensis_XP040158968     ESDEQIDFSGVDKVLAQPLKPQVPAEKSAKPAVPSTTAKPKAVTTGKQNVEILDQYDDDA     228
AnGambiae_AGAP006645         ESDEQIDFSGVDKVLAQPLKPQVPAEKSAKPAVPSTTAKPKAVTTGKQNVEILDQYDDDA     236
AnStephensi_XP035917013      ESDEKIDFSGVDKVLAQPLKP-VPAEKSVKTSPPSTTLKPKSTTTGKQNVEILDQYDEDA     226
IndInt_g22212                ESDEKIDFSGVDKVLAQPLKP-VPAEKSVKTSPPNTTLKPKSTTTGKQNVEILDQYDDDA     342
                             ****:**************** ******.* : *.** ***:.**************:**

AnColuzzii_XP040230463       DMDYQYNYDDDEDNDDDDDEDDDDDEEEDAETLPAQKITNKVNKEVAKKQTNPVKETKDQ     288
AnArabiensis_XP040158968     DMDYQYNYDDDEDNDDDDDEDDDDDEEEDAETLPAQKITNKVNKEVAKKQTNPVKETKDQ     288
AnGambiae_AGAP006645         DMDYQYNYDDDEDNDDDDDEDDDDDEEEDAETLPAQKITNKVNKEVAKKQTNPVKETKDQ     296
AnStephensi_XP035917013      DMDYQYTYDDDEDNDDDEDDDDD-DDDEDAEELPPQKAGKNVNSE-AKKQTKPVKKTKEQ     284
IndInt_g22212                DMDYQYTYDDDEDNDDDEDDDDD-DDDEDAEELPPQKAGKNVNSE-AKKQTKPVKKTKEQ     400
                             ******.**********:*:*** *::**** ** **  ::**.* *****:***:**:*
```

```
AnColuzzii_XP040230463       NKAAGKDNAEDYYDDDEYDD--DDNAEPSNSSYCPRGCICERNMHAYMVATCSRLDLETQ     346
AnArabiensis_XP040158968     NKAAGKDNAEDYYDDDEYDD--DDNAEPSNSSYCPRGCICERNMHAYMVATCSRLDLETQ     346
AnGambiae_AGAP006645         NKAAGKDNAEDYYDDDEYDD--DDNAEPSNSSYCPRGCICERNMHAYMVATCSRLDLETQ     354
AnStephensi_XP035917013      NKTAAKGTDESYYYDDEDDDDDDYNVDSSNSTYCPRGCICERNMHSYMVATCSRLDLETQ     344
IndInt_g22212                NKTAAKSTDESYYYDDEDDDEDDYNVDSSNSTYCPRGCICERNMHSYMVATCSRLDLETQ     460
                             **:*.*.. *.** *** **  * *.: ***:*************:**************

AnColuzzii_XP040230463       KFTSHITDLQVLDVGPKYPIELGPEFFKKIGLSHVVSIKITNCTIVYISPQAFAGLDELY     406
AnArabiensis_XP040158968     KFTSHITDLQVLDVGPKYPIELGPEFFKKIGLSHVVSIKITNCTIVYISPQAFAGLDELY     406
AnGambiae_AGAP006645         KFTSHITDLQVLDVGPKYPIELGPEFFKKIGLSHVVSIKITNCTIVYISPQAFAGLDELY     414
AnStephensi_XP035917013      KFTSAITDLQVLDVGPKYPIELGPEFFKKIGLSHVVSIKITNCTIVYISPQAFAGLDVLY     404
IndInt_g22212                KFTSAITDLQVLDVGPKYPIELGPEFFKKIGLSHVVSIKITNCTIVYISPQAFAGLDVLY     520
                             **** **************************************************** **

AnColuzzii_XP040230463       SVNLTNSGIDIIHPDTFANNTKLRLLTLSGNDLSAMQSVNHNTPYMDYMLKAPTVEELDI     466
AnArabiensis_XP040158968     SVNLTNSGIDIIHPDTFANNTKLRLLTLSGNDLSAMQSVNHNTPYMDYMLKAPTVEELDI     466
AnGambiae_AGAP006645         SVNLTNSGIDIIHPDTFANNTKLRLLTLSGNDLSAMQSVNHNTPYMDYMLKAPTVEELDI     474
AnStephensi_XP035917013      SVNLTNSGIDMIHPDTFANNTKLRLLTLSGNDLSAMQSVNHNTPYMDYMLKAPTVEELDI     464
IndInt_g22212                SVNLTNSGIDMIHPDTFANNTKLRLLTLSGNDLSAMQSVNHNTPYMDYMLKAPTVEELDI     580
                             **********:*************************************************

AnColuzzii_XP040230463       SRCKLQELQPNAFNELKNIIYINLSENNLSNLPEGIFDNVETIEELDLSMNNIVELPKNI     526
AnArabiensis_XP040158968     SRCKLQELQPNAFNELKNIIYINLSENNLSNLPEGIFDNVETIEELDLSMNNIVELPKNI     526
AnGambiae_AGAP006645         SRCKLQELQPNAFNELKNIIYINLSENNLSNLPEGIFDNVETIEELDLSMNNIVELPKNI     534
AnStephensi_XP035917013      SRCKLQELQPNAFNELKNIIYINLSENNLSNLPEGIFDNVETIEELDLSANNIAELPKNI     524
IndInt_g22212                SRCKLQELQPNAFNELKNIIYINLSENNLSNLPEGIFDNVETIEELDLSANNIAELPKNI     640
                             ************************************************* ***.******

AnColuzzii_XP040230463       FAKTSLAILHLKHNKITNNVDFVTADLQKLDLSFCQIRTVHNTMFKGMDGLTNLILKGNH     586
AnArabiensis_XP040158968     FAKTSLAILHLKHNKITNNVDFVTADLQKLDLSFCQIRTVHNTMFKGMDGLTNLILKGNH     586
AnGambiae_AGAP006645         FAKTSLAILHLKHNKITNNVDFVTADLQKLDLSFCQIRTVHNTMFKGMDGLTNLILKGNH     594
AnStephensi_XP035917013      FAKTSLAILHLKHNKISNNVDFVTADLQKLDVSFCQIRTINNQMFKGMEGLTNLILKGNH     584
IndInt_g22212                FAKTSLAILHLKHNKISNNVDFVTADLQKLDVSFCQIRTINNQMFKGMEGLTNLILKGNH     700
                             ****************:**************:*******::* *****:***********

AnColuzzii_XP040230463       IEKIKPMAFISLKNLRQIDLSYNNLEQISAQTFIGNKMLDIIRLNNNPRLKRLPNEGFEI     646
AnArabiensis_XP040158968     IEKIKPMAFISLKNLRQIDLSYNNLEQISAQTFIGNKMLDIIRLNNNPRLKRLPNEGFEI     646
AnGambiae_AGAP006645         IEKIKPMAFISLKNLRQIDLSYNNLEQISAQTFIGNKMLDIIRLNNNPRLKRLPNEGFEI     654
AnStephensi_XP035917013      IEKIKPMAFISLSSLRQIDLSYNNLEQISAQTFIGNKMLDIIRMNNNPRLKRLPNEGFES     644
IndInt_g22212                IEKIKPMAFISLSSLRQIDLSYNNLEQISAQTFIGNKMLDIIRMNNNPRLKRLPNEGFES     760
                             ************..*****************************:*************** 

AnColuzzii_XP040230463       SFNGTFTVYFMDVSNCDISELADNTFKTMPHLTRLNLAWNNLQTIRSTYFAHLNKLMDLD     706
AnArabiensis_XP040158968     SFNGTFTVYFMDVSNCDISELADNTFKTMPHLTRLNLAWNNLQTIRSTYFAHLNKLMDLD     706
AnGambiae_AGAP006645         SFNGTFTVYFMDVSNCDISELADNTFKTMPHLTRLNLAWNNLQTIRSTYFAHLNKLMDLD     714
AnStephensi_XP035917013      SYNGTFNVYLMDISNCDISELADNTFKTMPQLTRLNLAWNNLQTIRPPVMAHLSKLMDLD     704
IndInt_g22212                SYNGTFNVYLMDISNCDISELADNTFKTMPQLTRLNLAWNNLQTIRPPVMAHLSKLMDLD     820
                             *:****.**:**:*****************:***************   :***.******
```

```
AnColuzzii_XP040230463        LSNNMIINMDEKTFQNNRNLNTLNLAGNQIATLTSKLFHPLQYLSELDVSDCDLRTIWED    766
AnArabiensis_XP040158968      LSNNMIINMDEKTFQNNRNLNTLNLAGNQIATLTSKLFHPLQYLSELDVSDCDLRTIWED    766
AnGambiae_AGAP006645          LSNNMIINMDEKTFQNNRNLNTLNLAGNQIATLTSKLFHPLQYLSELDVSDCDLRTIWED    774
AnStephensi_XP035917013       LSNNMITELDEKTFQNNRNLNSLNLSGNQISSLASKIFHPLQYLSELDISDCDLRTIWDD    764
IndInt_g22212                 LSNNMITELDEKTFQNNRNLNSLNLSGNQISSLASKIFHPLQYLSELDISDCDLRTIWDD    880
                              ****** ::************:***:****::*:**:***********:*********:*

AnColuzzii_XP040230463        SAAGTKREEVLPNLKRLNVSYNEIMEVFVSDLESMGKLRVLDIRNNTLTCNERLPTLIDW    826
AnArabiensis_XP040158968      SAAGTKREEVLPNLKRLNVSYNEIMEVFVSDLESMGKLRVLDIRNNTLTCNERLPTLIDW    826
AnGambiae_AGAP006645          SAAGTKREEVLPNLKRLNVSYNEIMEVFVSDLESMGKLRVLDIRNNTLTCNERLPTLIDW    834
AnStephensi_XP035917013       SAVKAKREEVLPNLKRLNASYNEISEVFVSDLASMAKLRVLDIRNNSLSCNGRFPTLIDW    824
IndInt_g22212                 SAVKTKREEVLPNLKRLNASYNEISEVFVSDLASMAKLRVLDIRNNSLSCNGRFPTLIDW    940
                              **. :*************.***** ******* **.**********:*:** *:******

AnColuzzii_XP040230463        LQKKQISMGLGDTHDHVTHAEMTTELRTDNSATLKQWGQFAWEICQGGGSDSPNQRMTYS    886
AnArabiensis_XP040158968      LQKKQISMGLGDTHDHVTHAEMTTELRTDNSATLKQWGQFAWEICQGGGSDSPNQRMTYS    886
AnGambiae_AGAP006645          LQKKQISMGLGDTHDHVTHAEMTTELRTDNSATLKQWGQFAWEICQGGGSDSPNQRLTYS    894
AnStephensi_XP035917013       LQQKQISMAD-NSHDHVTHAEMN-VLRTDNSVSLKQWGQFAWEICQGGSG-DPDNTLSYS    881
IndInt_g22212                 LQQKQISMGD-NSHDHVTHAEMN-VLRTDNSVSLKQWGQFAWEICQGGSG-DPDNTLSYS    997
                              **:*****.  ::*********.  ******.:***************.. .*:: ::**

AnColuzzii_XP040230463        ESDEEYNIEDEDDVSEAADADVMAEAKNAKSNKIVDSKNELDDYSDDSVDSDDSEDEDSA    946
AnArabiensis_XP040158968      ESDEEYNIEDEDDVSEAADADVMAEAKNAKSNKIVDSKNELDDYSDDSVDSDDSEDEDSA    946
AnGambiae_AGAP006645          ESDEEYNIEDEDDVSDAADADVMAEAKNAKSNKIVDSKNELDEYSDDSVDSDDSEDEDSA    954
AnStephensi_XP035917013       ETEEDYDEDEEDDSED-ADAEV-TADTNAKSNKIVDSKNELDEYSDETADSDSDDNDNAD    939
IndInt_g22212                 ETEEDYDEDEEDDSED-ADAEV-TAGTNAKSNKIVDSKNELDEYSDETADS--DDNDNAD    1053
                              *::*:*: ::*** .: ***:* :  .***************:***::.**  .:::::

AnColuzzii_XP040230463        --DEDD---EEDDEEDDDDDDDDEEEQERENILDAANGIQKIEQGILTGKQADED--DDG    999
AnArabiensis_XP040158968      --DEDD---EEDDEEDDDDDDDDEEEQERENILDAANGIQKIEQGILTGKQADED--DDG    999
AnGambiae_AGAP006645          --DEDD---EEDDEEDDDDDDDDEEEQERENILDAANGIQKIEQGILTGKQADED--DDG    1007
AnStephensi_XP035917013       ADSEDDEDDEDADDDDDEDEEEEDDVQERENILDAANGIQQIEQDILTGKLNNNADDDDD    999
IndInt_g22212                 ADSEDDEDDEDADDDDDEDEEEEDDVQERENILDAANGIQQIEQDILTGKLNNNADDDDD    1113
                                .***   *: *::**:*:::::: **************:***.*****  ::   **.

AnColuzzii_XP040230463        EYE--DDGASVDDVVILENGSMFAVSSVWIVVACILFGISMLLLVVVKVLTEVMKRRGER    1057
AnArabiensis_XP040158968      EYE--DDGASVDDVVILENGSMFAVSSVWIVVACILFGISMLLLVVVKVLTEVMKRRGER    1057
AnGambiae_AGAP006645          EYE--DDGASVDDVVILENGSMFAVSSVWIVVACILFGISMLLLVVVKVLTEVMKRRGER    1065
AnStephensi_XP035917013       EYEDDSEGTNVDDVVILENASMFALSTVALMVSCIVIGSLIMVLLVAKIVGLIFKRRGER    1059
IndInt_g22212                 EYEDDSEGTNVDDVVILENASMFALSTVALMVSCIVIGSLIMVLLVAKIVGLIFKRRGER    1173
                              ***  .:*:.*********.****:*:* ::*:**::*  :::*:*.*::  ::******

AnColuzzii_XP040230463        YRQALLASKNSFVYQKLTEDIVPSKVPKIHRYEPINQV    1095
AnArabiensis_XP040158968      YRQALLASKNSFVYQKLTEDIVPSKVPKIHRYEPINQV    1095
AnGambiae_AGAP006645          YRQALLASKNSFVYQKLTEDIVPSKVPKIHRYEPINQV    1103
AnStephensi_XP035917013       YRQALLASKNSFVYQKLTEDIVAPKVPKVHRYEPINQV    1097
IndInt_g22212                 YRQALLASKNSFVYQKLTEDIVAPKVPKVHRYEPINQV    1211
                              **********************  ****:*********
```

*Supplementary figure 4b : MSA for IndInt gene g22212.t1 across other Anopheles species shows high conserved homology.*

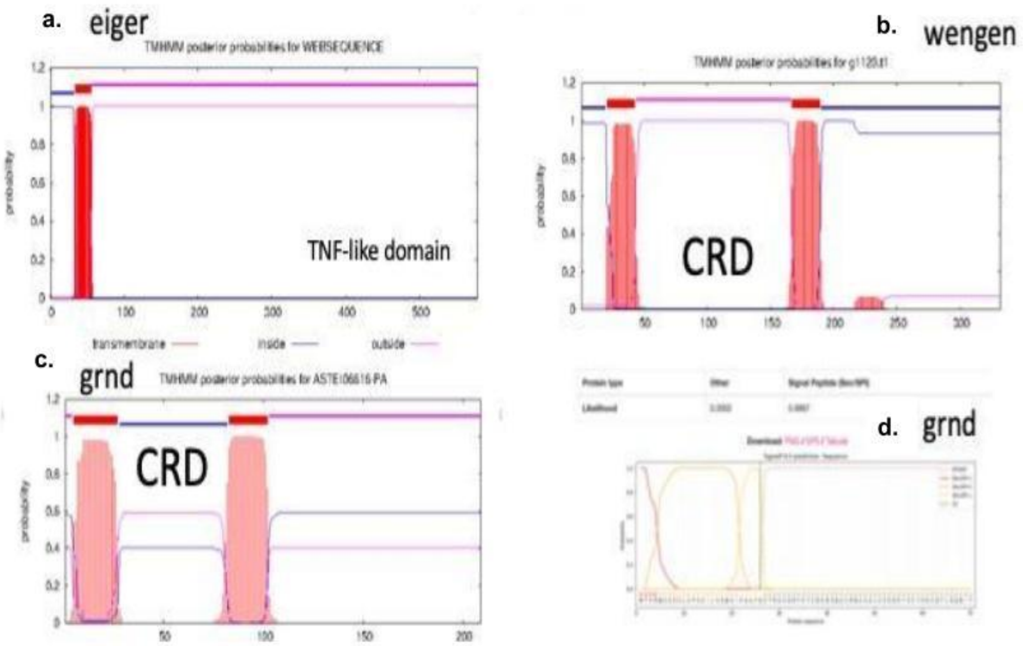


*Supplementary figure 5: (a) transmembrane prediction for eiger (b) transmembrane domain prediction for wengen (c) transmembrane prediction of grnd (d) signal peptide prediction for grnd.*

## UCI

```
# WEBSEQUENCE Length: 475
# WEBSEQUENCE Number of predicted TMHs:  0
# WEBSEQUENCE Exp number of AAs in TMHs: 0.00271
# WEBSEQUENCE Exp number, first 60 AAs:  0
# WEBSEQUENCE Total prob of N-in:        0.02124
WEBSEQUENCE     TMHMM2.0        outside      1   475
```

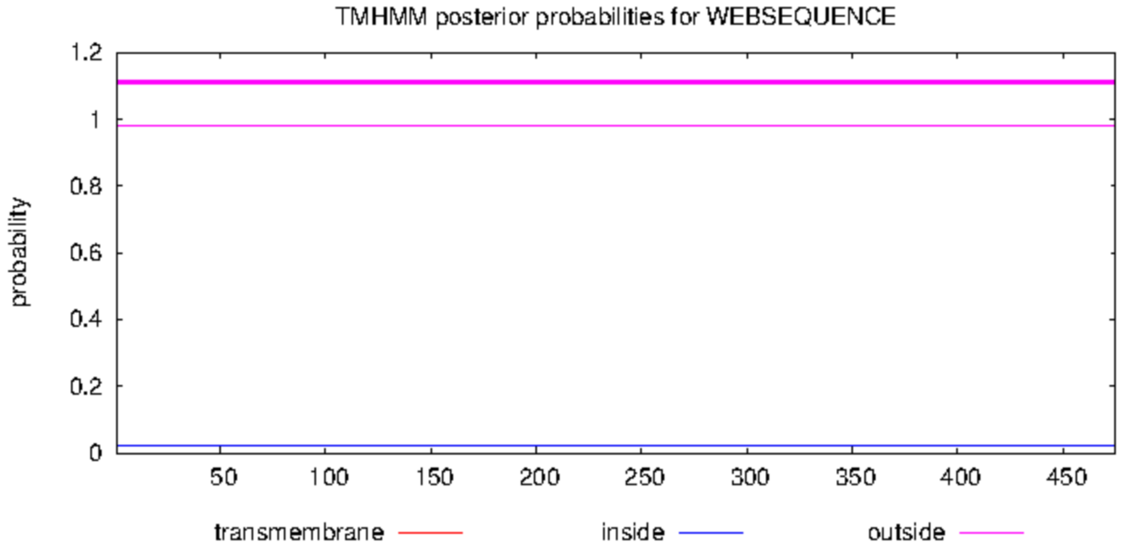


## STE2

```
# WEBSEQUENCE Length: 455
# WEBSEQUENCE Number of predicted TMHs:  0
# WEBSEQUENCE Exp number of AAs in TMHs: 0.00349
# WEBSEQUENCE Exp number, first 60 AAs:  0
# WEBSEQUENCE Total prob of N-in:        0.02710
WEBSEQUENCE     TMHMM2.0        outside      1   455
```

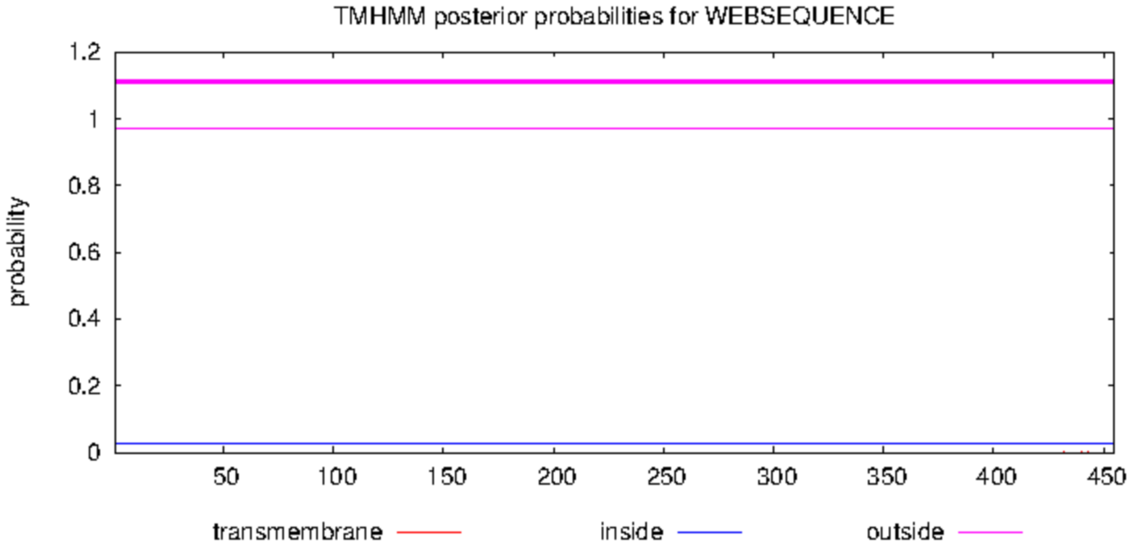

***An. gambiae:***

```
# AGAP006771-PA Length: 544
# AGAP006771-PA Number of predicted TMHs:  1
# AGAP006771-PA Exp number of AAs in TMHs: 22.71772
# AGAP006771-PA Exp number, first 60 AAs:  22.71771
# AGAP006771-PA Total prob of N-in:        0.99203
# AGAP006771-PA POSSIBLE N-term signal sequence
AGAP006771-PA   TMHMM2.0        inside       1    32
AGAP006771-PA   TMHMM2.0        TMhelix     33    55
AGAP006771-PA   TMHMM2.0        outside     56   544
```

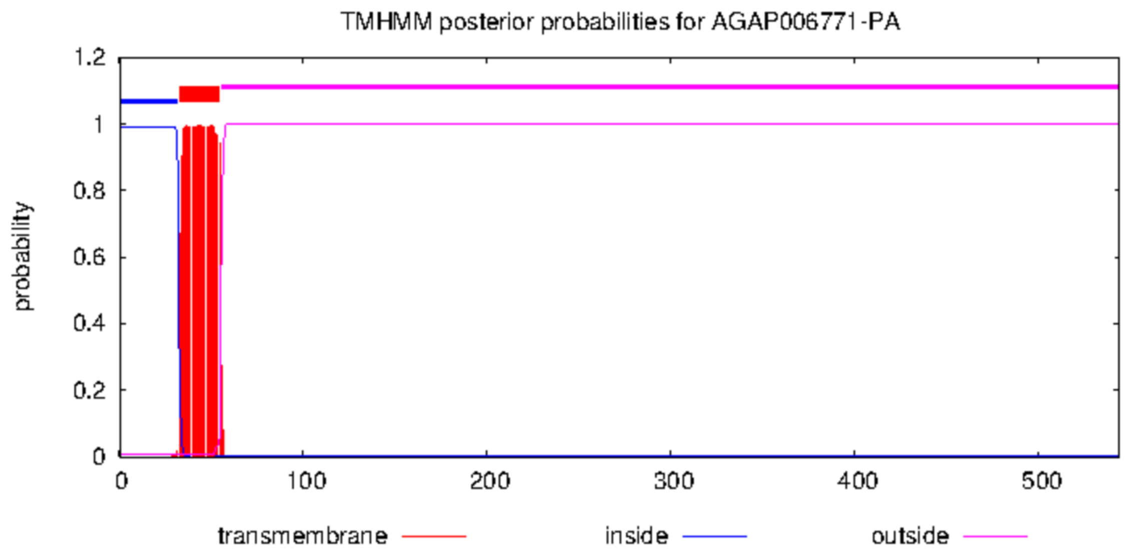


*Supplementary figure  6: TmHMM analysis of the eiger gene in the UCI and STE2 strains and the An. gambiae PEST strain, respectively.*

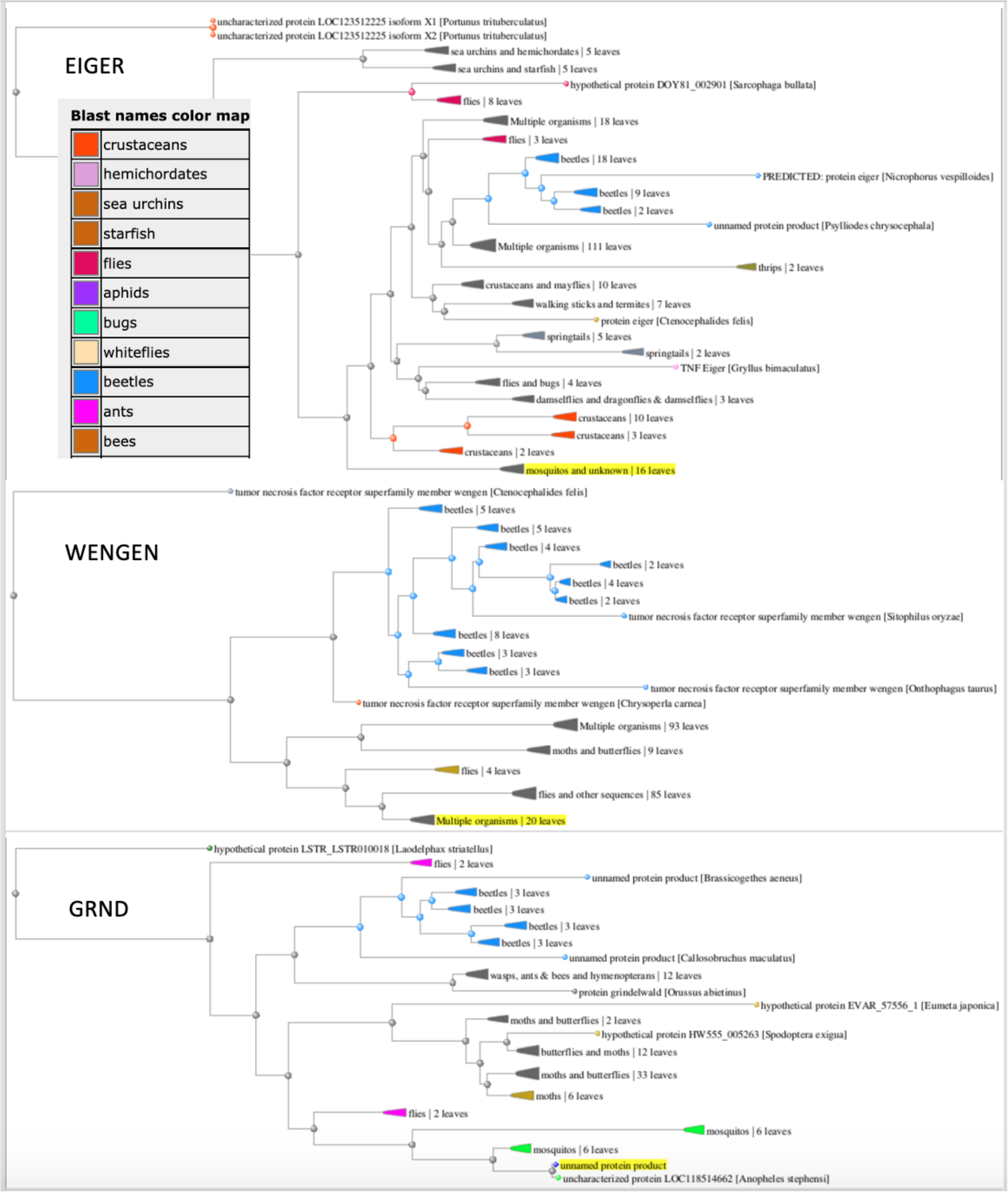


*Supplementary figure 7: BLAST result of eiger-wengen-grnd proteins from IndInt against NR-protein database at NCBI suggesting diversity in eiger gene across vectors across arthopods.*

*******************************************************************************

**>ANSTEP-UCI_TRAN_00009302-RA protein Name:"Similar to litaf Lipopolysaccharide-induced tumor necrosis factor-alpha factor homolog (Xenopus tropicalis OX=8364)" AED:0.00 eAED:0.00 QI:437|1|1|1|0|0.33|3|1007|126**

MTKDGPPPYGFVAPPSAPPSYAQAVGGVPPSSPFTPQQPVLTSAQIVTTVVPIGPQSTHMVCPSCH
SEVVTKTTTSPGMIAYVSGFLIALFGCWLGCCLIPCCIDECMDVHHTCPHCKAYLGRHRR

**>ANSTEP-UCI_TRAN_00010133-RB protein Name:"Similar to Litaf Lipopolysaccharide-induced tumor necrosis factor-alpha factor homolog (Mus musculus OX=10090)" AED:0.14 eAED:0.14 QI:206|1|1|1|0.66|0.5|4|598|158**

MNPSGKGSGPEGFQAQPLNQPPYPAQSPPYPGQMPAATGGYPHPSANVPNYAHPPPPPPYDANSNV
IPPPQNAGTTYVQVVTSPQVGPDPASMVCPSCTKHVITRLDYETSTKTHIAAGLLCLFICWPCFWI
PYIIDSCKNANHYCPNCGAYIGTYRG

**>ANSTEP-UCI_TRAN_00010132-RB protein Name:"Similar to Litaf Lipopolysaccharide-induced tumor necrosis factor-alpha factor homolog (Mus musculus OX=10090)" AED:0.01 eAED:0.01 QI:206|0.5|0.66|1|1|1|3|787|124**

MDPPPYDQINRPVPAAYPHQQTPTADPFKSASLHTQEPPSLQTTVIVTSPQVGPDPTTIICPSCRA
TVVTRLEYETTTKTHLCAGLLCLFLCWPCAFVPYCSTACRDANHYCPNCGSFIGTYRK

**>ANSTEP-UCI_TRAN_00010131-RA protein Name:"Similar to FV3-075L Uncharacterized protein 075L (Frog virus 3 (isolate Goorha) OX=654924)" AED:0.00 eAED:0.00 QI:483|1|1|1|1|1|2|936|82**

MTTIIVTNPQVGPDPMTITCPSCRATVRTKVKHESTTSTHACALLLWIVCWPCLCLPYCC
NSCRDANHYCPRCNTFLGSYKH

*Supplementary text 1: FASTA sequences of the LITAF genes from the UCI strain*

**>g6300_ADAM17_TACE_IndInt**

MSLPSIMLTVVLVVSFAVLIVPLQGQLHKNLKYYETLHAKDLSHRIEKRGTKHSTHPFNTIKEVEF
KVLGRKFRLILHPHASVLHSNFRAYSVDGNGSESIVHLDRSNFFKGRVFGEMESHVNAHIDDGVMT
ASVVLPDETYHIEPSWRHLPHLSDKHMIAYRTSDIKFSWDQVDAISGDLGVPRTCGYVKEGLELEG
QPDDGDEAADGGQAFADGGVDAYENDRDPTPETVWHAEDSQDARKSRRKRQADQYEYTPTKTRCPL
LLVADYRFFQEMGGSNTKTTINYLVRAPPPTGSFGAPFVVPFSNRFLPFYQISLIDRVHKIYNDTI
WQDRSDQEGFKGMGFVIKKIVVHSEPTRVRGGEAHYNMVREKWDVRNLLEVFSREYSHKDFCLAHL
FTDLKFEGGILGLAYVGSPRRNSVGGICTPEYFKNGYTLYLNSGLSSSRNHYGQRVITREADLVTA
HEFGHNWGSEHDPDIPECSPSASQGGSFLMYTYSVSGYDVNNKKFSPCSLRSIRKVLQAKSGRCFS
EPEESFCGNLRVEGDEQCDAGLLGTEDNDACCDKNCKLRRNQGAVCSDKNSPCCQNCQYMMAGVKC
REAQYATCEQEARCTGNHAECPKSPPMSDGTMCQERGQCRNGKCVPYCETQGLQSCMCDIIADACK
RCCRQSINETCFPVEPPDVLPDGTPCIQGFCNKGMCEKTIQDVVERFWDIIEEININKVLRFLRDN
IVMAVVMLTALFWIPVSCVIAYFDRKKRKEDWKEYEWSQKLDLIHPSDRRRVIHIRVPRQKITVAR
M

**>g22826_Eiger_IndInt**
MTAETLKPFLNLPNATANDLKAHCAQRSTVKRGLVALGCILAAALCCGLIGVQIWHLNKTAILQQE
VDDLKLQLYLRAREFTDYEASASARPSSLTITTPDRMIMCADGVRRTPVESQRDLFMMGLVDACKN
IQTLVSLICPSFDNELFDSPDEYVYEPEDLDPDVSDDGHREQETNESGLSSSGMQGDDLELDDSEE
KENLGIEDLRETNDDDDDDDDIIIDDRVDIGVPAGGKRRARSISGVTRQGVPIVDEPYVPRRNRTR
HPHRIFEQLRQRPEELVTPPTSAEMFRWDVDSRKTSHTAARHQSYGSISIRPYQHEAHSLREHHST
TPMPVAYRSHGGVRHHSRNELHAGNPTKPPAQILNRMSRVQATGDSMRKQLQQTQRFPKVVGNPGT
QKEIVMAPESRVRLRQRKGGAVQPAEPVAKGVHLVELSTMDAVHSDSRYQEWSANDNASKQAIQSS
SFAFDGERLTVNEPGLYYVYAQVTYSNEFEANGYKVLINGRKHLSCTVNTAQQGENTNTCFTAGLA
EIAHAGTTIAVEDVAHGRQHVMYPEKTFFGAFKIGRLAAPTVTHRRARKVA

**>g1129_Wengen_IndInt**
MTHVPDIRCGMAQVGRRRSHPRTGRGVIPTTTLVLLAILLQLMDLGDGPGGGRSMLVRAACESRKS
WWDPTLGECAPCLICADHQVVLRPCQDYMNTVCGTMKDLTASVSRPYPHLPEGNGNGIGSVGRTRA
HHWKEERRKEGDGVEGYRRVVPAISTEEILWDWQVASLLIAIIGCLLFLLAAGCVALNQSRQWRRI
EKHFDAAQNISKPINNERAPYGILAVGFAVVDMEALSAQLVNHLASMQHLESGPILLENFDHGRLR
TTGHHPIEVRCVYLDQLLDEKCSQKAHSVQPTAGNLYIEESIDTPRLQSPIPSARSPPPPPISLIR
HY

**>g18030_Grnd_IndInt**
MTVRWTSSVLVPVVILVAWSIPSAFGCTRKCDRFEYCDENSNACRNCSALCEKDEYSCYHKCQTLR
QNITTLQESVLTMKTIVLVFSVVAFVLICTALALLIKKYVHCIREFCRWRPQNKNTTTPPVAYTHE
NPNTKSPKTVPKNGANGPKQTSTSVSIYPETEVDHSVQTGTTSISHRYPAEDSTESYSYDNAACNV
TPTSNNPMPKF

**>g5130_TRAF2_IndInt**
MSRPVKREVSAEENCLQSAAKVDDDLLEARYECPICSCWLNEPILTKCGHRFCRKCITDWLNGKNS
ICPLDNEPLDIKCDIFPDNCTRREISQIKKPCPNSIRGCVDQFSPTEIDSHLRQCPFAMSRQSQPC
PFARIKCKFIASDEDALNAHIASDCQQHLQLLLETYTGSNDRYKFWDPPAKNSVPEMSTNELVRSM
YERIVILEQEVHILGIKLSKQELQLTKINQEVDPRYSGGVLLWKLEDFSNKIDSMVANSNCMFYSG
QAYTSPHGYKFCARINVSPRTKDSIGLHVHLMQSENDYHLEWPFKGRIKITLLNVRSPELSQHDTI
MSKPEILAFHRPHQDISPRGFGFLEFAKIKEILAKFADNNTVVLKIQMNIV

**>g13550_TAB2_IndInt**
MCSDLGRYDYQFSIRLRVATAKGAAVRTNCGWLREENERTENRESERSDEAEFDDRTVLRCDGEKK
DDFVSGYQGVWWWFLQTPAIPSTTQSNNTQHYYDEPATAVFTSNSSAATSGSGSNAADSSSILKQR
ATCACSNISIMQLFHEMKQKYPTVPDTVVSELVTQNCHDRPACIGKLEEAVLGTPAQTTYPSQSIH
SGSLKRRSGDRKLAGNRSDGSNSYSSSNSSSNSSRESSVDSSRLQSASYPTTHQQQQQQGVPTTGG
GTGYSDNRFIGESRISSSCANRPTTLAVRPAAAGTGVGNFGATPPPPPMRPNRIAPPCPPNNVPLG
TSTITTTTTASSSTELGETVNLQVNVTVSPKAGGPMIAGQRHTSTISLQPEPPYSRELAQVSSTFS
GGNPLVMGSPTAGTVAGSTPGGSSRTGSASVGIGAGGNGGRSSTSVNLTLRQPTDGRPRTPIHIHA
SPLKYTAKNFNAQSGIQSKLEVTFRDGFGSFSAMRAQVPGYEPQREMMASPQQPEREPCAVALPDG
HSGWYQSPSSAGSRVPYGGLSGFPPPLSSSSPLPNLAAHQMVSSRGMNGQSLFADGQLENDRLTKE
MRYQNFVVAEMAVSQQLEQKQRLSLEVERKRTQFESICREIFVLQQPLRFIDAELLDREVLVLAAE
VEQLQKEVDSCDEEEARIQAAAAAAATGSSITDGLSALSVEGGSPLMLPPSMVSGASGGGTVVNRP
PRPPRPPPPRAPTQSPSRGSSGSVTPSTPHLAPSLAVIGGTSSPVPTPTSSVSSSCSTSSSFQSNG
GGVGDPTAPNRLQPSATTGPNQPWTCSLCTFQNHELMPACEVCSLPKASGSRTAVAAPMTNSNPAF

AVAGDVRDGVGGSPHAAGVLLRRQHHLSVDTGVAAAAAAAAAAAAAAAVVGASVAGPSTAPPYPGVA
EMPPPTFASLTTGHTSTSMPLQPIPLSHGQQQQQQQQQQQQQPTQTVQQQSQQSNPPPPLQNAQYQK
SSIEC

**>Intsfg5795_Hep_IndInt**
MSDDQSSLSSPATTPSSARPFMPLPLELNGERRKPSKKLSFQGCQGGQAPMIPDPTRERIRMQAAA
GKLQIAPNQTYDFTSDDLLDEGEIGRGAFGAVNRMKFTHTGTVMAVKRIRSTVDEKEQKQLLMDLE
VVMKSNDCNTIVTFYGALFKEGDCWICMELMDTSLDKFYKFICECQQSRIPEPILAQITFATVRAL
NYLKEELNIIHRDVKPSNILLKRNGDIKLCDFGISGQLVDSIARTKDAGCRPYMAPERIDPQRAKG
YDVRSDVWSLGITLMEVATGKFPYPKWGSVFEQLSQVVEGDPPRLCTTYNGMEFSIDFVNFVNTCL
IKEERDRPKYGKLLQHAFIQHAEKSDTDVAAYVSEVLESMANNGITQFTTNLPAEGWNESFN

**>g5319_MKK4_IndInt**
MSDDQSSLSSPATTPSSARPFMPLPLELNGERRKPSKKLSFQGCQGGQAPMIPDPTRERIRMQAAA
GKLQIAPNQTYDFTSDDLLDEGEIGRGAFGAVNRMKFTHTGTVMAVKRIRSTVDEKEQKQLLMDLE
VVMKSNDCNTIVTFYGALFKEGDCWICMELMDTSLDKFYKFICECQQSRIPEPILAQITFATVRAL
NYLKEELNIIHRDVKPSNILLKRNGDIKLCDFGISGQLVDSIARTKDAGCRPYMAPERIDPQRAKG
YDVRSDVWSLGITLMEVATGKFPYPKWGSVFEQLSQVVEGDPPRLCTTYNGMEFSIDFVNFVNTCL
IKEERDRPKYGKLLQHAFIQHAEKSDTDVAAYVSEVLESMANNGITQFTTNLPAEGWNESFN

**>Intsfg11051_PI3K_IndInt**
MKQEQTNTTMKRTAHSTLPGNDPSWQVPHRIYLQQQHQQHLHQQQQQQPMQQRPLFDYEFWKNPDE
LTELHFLMPNGVMITMCIPIHITLEELKTDVWEEAERYPLYCHLGDKNKYCFSTLASGRTNCNTTH
ENEATRLMDVQPALGILRFVERTNVSEDTKLMENISILLDSKLPMKTLVNPEVNDFRAKMSLLSEE
IGSRRAAMTRMERIAYQYPAKLVSGSHVPDQISRRLANVDGHFCVVAISTDMQVTVKVPCRATPDE
VLNRILEKKRISMKARMDNSSDFILKVCGREEYIYGAYPMISFQYVQDCLSRDETPTFVPRLVRSV
EVFKNDIYDARDDFTQWSSTTATTNTTSSALVSTSSSVSTMSLVSVGKHNSSIASTGSSSVYSSSS
QSQQSHTLRKVKYVTSWEVDTKLQCTVQEIRGLNIESDKELGVQLGLFHGGKSLCKTARTRTVTVN
SGKAVWNETICFDINVSNVPRMARLCLVVYENMRTTKSTGIRTRRTKDGLINPIAWVNTMVFDYKN
QLKSDSVTLYTWDYAEDAQSEDILHPLGTVEPNPNRDNNMFVSMLLSFGPYGPEGRIIVYPGEEEL
LAHAAKMSHRNEHLNRESAEDTRSIKAIMSAYMYNDRLNDIHEQDRNAIWAKRRECMLQMPQGLPC
LLYCVEWNNRDEVSEIVSLLQEWPKLLIERALELLDYAYADKYVRRYAVDCLRTIEDDELLLYLLQ
LVQALKHESYLNCDLVYFLLQRALHNQHIGHYLFWHLRSELSVPSVQVRFGLILEAYLLGSPEHVG
VLLKQMQCLRYLQICSDNVKKGSKEKGRALLMEQLAKEGHLTSDLISPLNPSFRCKAVRTERCKVM
DSKMRPLWIVYENSDPNGDDIHMIFKNGDDLRQDMLTLQMLRIMDRIWKSHGFDFRMNPYSCISTD
HKLGLIEVVLNAETIANIQKERGMFSATSPFKKGSLLAWLREHNNTDELLAKAIQEFTLSCAGYCV
ATYVLGVADRHSDNIMVKKTGQLFHIDFGHILGHFKEKFGFRRERVPFVLTHDFVYVINNGRTDRE
AQEFCHFQTLCEEAFLILRQHGCLILSLFSMMISTGLPELSSEKDLNYLRETLVLDKTEEEARTHF
KHKFSEALANSWKTSLNWASHNFSKNNRQ

**>g17575_NIK_Brun_IndInt**
MLSQGPGDPIMAHPDYEQHHYHHGCLLILVRGIGSSKPRSLQRVFERVQRVNNVKIPADSTGTPRD
IWVRYIRDHPVENNDWGDFQTHRRLLGLITVGKFEAQSELNELCRVHESLKVKYTHTLSRCLLFGP
STDELQKLNGAAAGTAVAPDGGSKATLEKCFQTPSNFKSRAFFYPENDPCSNLETKISEFITSIYY
ILELKRMEKTREKLEKAPLLLAPFEKKDFVGLDLESRNNKKRCIGRMTKHLGDLTLQAGLVAESLN
FFHAASETLRAISDSLWLGAANEGLCAASAILLYPNFRYTMSIQRNSSLQENSSSPQKFNLARNQL
YTGSYNGADSSTLKHKKSDMVINLSASDTATILGADKTSASSNSSASSISSSLSSGSGGSGTASGS
SSSDSGTLVNRAGSGVAAKFPSNILQPDEITVRYRDAIINYSKYRNAGIIETEAALKAARICIEQG

KNLDVAMFLQNVLYINLNMTEQQRVRRFEVLTDLYQKIGYNRKAAFCQRLAAWRHVAQSNSNPDWG
QSYRLMLESFSGHKLSLEPNEVLENNMGWPVLQIDLLQQLVGTARRLGQSALATRHMTFLLQTMWK
HLTAQEQREMALQLQNLSAQCEGAPVPLVLENGIVIPPANLTDLPHCSQLLVKDLAPHLKPVKIVV
NKVDSGPFLFTPIHFSSLDRRGIEKDDSKITFNWVQHDVCEVSVSLMNPLPFELQVTDMRLLTTGV
VFEAFPQTVTLQPNVSTNVSLHGTSIECGELEIQGYSTHTLGVKSNCRLKHMLHRRERHLPPCYKV
KVIPALPKLETKTSLPQTATFSGMPNADFVTTSASITLYNGERGECTITLTNSSNIPIEYVDATFH
STLEASLQSRIFQLATDELHRKLPIQPNASIDFKLVIFGEADFLGALTAGPGQTAGYSLPHNPDGM
NSAGPQSLTVSHGGGGGPLGGGMLSAGGGSGNPSIPSRISSPTNTHRRNELLTSSFRSSHSGHSSL
ATLSVGISTGHVPRQLDAQLRFKYSGGEGLQEGFCRQCAISFNVELLPSAQITNWDVLSAEIPSQF
YLVLDVVNLTAQEMSLNYTSNKTILIEAKESCRVPVPVQRCPLERIFAAVAEHQQQHHNHLHQHHH
HHSIGSGSADHHNMPSILSGGAGTSTSDSSDLTERVCSEHISENVNLKWSLPGIDCHGTASLRGIT
LSPAMLDLVTVPPLEWEVKVDDQVVAPQSEVTCVTGQFLSFSISICNLSASVLHQVQLSVQFYQDY
QNGVQNYRLETRVTMSGPNHILIPSLNKDEKAFHKCSVLFFT

**>g8096_AIP1_IndInt**
MHPERNPDALAKLHKNGFEKVEIAVYNVLAREFIYATLPRTQRGQPIVLGGDPKGKNFLYTNGHSV
IIRNIDNPEIADIYTEHSCAVNVAKYSPSGFYIASGDQSGKIRIWDTVNKEHILKNEFQPIGGPIK
DISWSPDSQRIVIVGEGRERFGHVFMAETGTSVGEISGQSKPINSCDFRPARPFRIITGSEDNTIG
VFEGPPFKFKMTKQDHTRFVQAVRYSPSGHLFASAGFDGKVFMYDGTTSELVGEVGSPAHSGGVYG
VAWKPDGTQLLTCSGDKSCKLWDVETRTLISEFPMGSTVDDQQVSCLWQGNHILSVSLSGFINYLD
VNNPTKPLRVVKGHNKPITVLTLSDDRSTIYTGSHDGAVTNWNSGSGTNDRVAGVGHGNQINDIRA
AGDFVYTAGIDDSIKQISVEGNTYTGVDSKLACQPRGMDILKESNTIVVGCVKDITVLQDNRKVSS
VPISYESSSVSINPETMDVAVGGDDSKVHVYTLHEGQLTHKLDLEHLGPVTDVRYSPDNKLLVACD
ANRKVILYSVAEYKPPHNKEWGFHNARVNCVAFSPNSELVASGSLDTTIIIWFVNKPAQHTIIKNA
HPQSQITGLVWLDNETLISTGQDCNTKVWNIENVA

**>g24355_IAP_IndInt**
MDHQYDKGSLLLCLAIFVHKLTIVFHWTQNYLKVLALARVQQRKAGGEVVSSSESAAPSSTPKAPG
DADDDMREMAHALSLPAPIDLPDNKQKDDDLACMSPEYFHIEENRLRTFGRWPVAFISPNVLARYG
FFFVGTDDTVKCYFCRVEIGLWEPQDDVIQEHLRWSPFCPLLKKRPTNNVPLNANYLDAVPEPSYD
TCGISVRQHSYAENVNDRARPDLDRMSGDSWSGASDISLSSSGSSSHNGGDAEPMSGVSGLSSGGG
GVGGGGGLQQDRASMTAAEWNNGVLMGEHSLMRRPEYPNYAIEADRLKTYEDWPTSLKQKPQQLSD
AGFFYTGKSDRVKCFSCGGGLKDWEQDDEPWEQHAIWYSNCHYLQLMKGREFIEKCNELKEAAASA
TSGTSSSMSSASSQPSTSGISSASSSVMSTSPASSSGFSSPTPAADEERTLRCTDHYSSGSDEGGE
DDAGHNRKVPSDGKICKICFVNEYNTAFMPCGHVVACAKCASSVNKCPLCQQPFINVLRLYLS

**>g17640_JNK_IndInt**
MSSRHAFYTVEVGDTKFTILKRYQNLKPIGSGAQGIVCHTKAKDRTKPLTKEPRQGQPPPQQQQQQ
QHTVANMNRRANNYVRVQFGDTEFEVPDRYINLEAKGFGAQGTVCAAYDTVTQQNVAIKKLSRPFQ
NVTHAKRAYREFKLMKLVNHKNIIGLLNAFTPQRTLEEFQDVYLVMELMDANLCQVIQMDLDHERM
SYLLYQMLCGIKHLHSAGIIHRDLKPSNIVVKSDCTLKILDFGLARTAGTTFMMTPYVVTRYYRAP
EVILGMGYKENVDIWSVGCIMGEMIRGGVLFPGTDHIDQWNKIIEQLGTPSQSFMARLQPTVRNYV
ENRPRYTGYPFDRLFPDVLFPTDSNEHNRLKASQARDLLSRMLVVDPEHRISVDQALVHSYINVWY
DESEVNAPAPGPYDHSVDEREHTVEQWKELIYQEVMEYEARNNLADATDEAMAAAAEAAEASSTTA
AAAAAAEDLQPPSEEPPDAMSPAEE

**>g11102_p38_Bsk_IndInt**

MANFYRTEINKTEWEVPDKYQALTPVGSGAYGQVCSAMDTVHNVKVAIKKLARPFQSAVHAKRTYR
ELRMLKHMNHENIIGLLDVFHPGDNKLESFQQVYLVTHLMGADLNNIIRTQRLSDEHVQFLVYQIL
RGLKYIHSAGIIHRDLKPSNIAVNEDCELKILDFGLARPTENEMTGYVATRWYRAPEIMLNWMHYN
QTVDIWSVGCIMAELLTGRTLFPGTDHIHQLNLIMEILGTPNDEFMAKISSESARHYIKSLPKTEK
RNFSDVFRGANPLAIDLLEKMLELDADKRITAEQALAHPYLEKYADPSDEPTSSLYDQSFEDMDLP
VERWKELVFKEVLNFVPQQHAHIGGEPQA

**>g21984_AKT_IndInt**
MTATDNTVRFSDHTLDKATKAKVTLENYYSNLITQHGERKQRQAKLEASLKDETLSESQRQEKRMQ
HAQKETEFLRLKRSRLGVEDFEALKVIGRGAFGEVRLVQKKDTGHVYAMKVLRKADMLEKEQVAHV
RAERDVLVEADHQWVVKMYYSFQDSVNLYLIMEFLPGGDMMTLLMKKDTLSEECTQFYIVETALAI
DSIHRLGFIHRDIKPDNLLLDARGHLKLSDFGLCTGLKKSHRTDFYRDLSQAKPSDFIGTCASPMD
SKRRAESWKRNRRALAYSTVGTPDYIAPEVFLQTGYGPACDWWSLGVIMYEMLMGYPPFCSDNPQD
TYRKVMNWRETLIFPPETPISEEARDTIVKFCCEAERRLGSQRGIEDLKLVQFFRGVDWEHIRERP
AAIPVEVRSIDDTSNFDEFPDVALEIPAHPQPEGEVLKDWVFINYTFRRFESLTQRARAADTEAKQ
QQQQHIVSPNPQSIDRPTNGMMAQDRVTG

**>g17088_IKK_IndInt**
MSGLHSATFIVRGCAVNRLNIAQPAAFFRYIKTQLSVLCALLQESNRGVSLLVNVLANMIKPFEDP
PLIGDWCREKRLGNGGFGVVSLWRNKQTQQAVAIKKFHILQDRSEITDKHCERWRNEVKLMTETVQ
NENIVRTVNVQPTSFIQELLRSSANGLPVLCMEYCEGGDLRRVLNRVENCSGLREQDVRDVLRSLR
NAVAYLHSLKITHRDIKPENLVLKQQGERFIYKADLGYAKALDKQSLNASLVGTVEYIAPDLIYCD
RYNCSVDYWSMGVIGYEIITGVRPFIPHAPITRWMMHVQQKKSADIAITEDNRENYTYHTDIFPEN
HISDCLRKELESWFRLALEWNPKKRGYVPYTKAEPSLANGENGTKGVKQTEDNKPSTVLKIFSLLD
QILEKRILVLFSLYDCRWIDLEVTPETGMETLRDHVYRVTGIPVDDIEFVLPLEQKQPAVGNDTRP
YDLYLPDFYGKPMVYVVHRGSRDSIVQRDLKPRIPKSITDVFQNIKVKLKPHMLRQFIANSYYFIA
QEQRLYGQVLDGIRNYGLMLNDNIARRKDEIGRMNKIVYAILGGVEYHKLTVCHARDALNVERRIP
SATFEMASKRWIENGARIESNVRKLAEMADTITKRYESVLKRSRDALRHALLHPQLDQHDTFGLRN
VECRYEQTRARLMEKILNEKSHMDMSQAVYECLKQRDVLLRELGFLELQQQILDVRREIQEIEKVV
CKVIDVTEKYKRDLARLKLEHQDEVWKMLSDYGRSAAAGSCENGVVHLDNILPLGVAHTDPPVINK
PNFLVGGPTTPQLQSTVSNESFDANQVCCLGYTDPNVEDLIAANETLIHTTTDLLSNSFSLLKLAD
GAE

**>g13192_Caspase1_IndInt**
MEEFALSASLRHSTDELDANAVNRSTNSAIPSALSTSNDVTAQGSDHYDTSHPRRGIALIINQVNF
KDLRKRDGSDKDRDCISAALQGIGFEIRTLDDPNRKELLAMLEKVANEDHSQHDCLVVVVMTHGKE
NNFLYARDKSYEADRLWEPFLGNACPSLRGKPKLFFVQACRGKQLDQGVRLVNMSIDENVSVDSVP
EPVSYVIPTMADLLVMYSTYDGHYSWRNPVNGSWFIQSLSIVLTATAHREELLHILTAVSRRVAFH
YQSDVPQNVKMDAMKQMPCIVSMLTKLLYFPKKS

**>g22865_NFkB_IndInt**
MSLDHCSIALDVLLLQLQQQQQQQQPPPRLDVPYSLQTEQQQQLLGASPNQYTILSMDAPSAAAA
VVSVGDYTLGSSRSYASALSPSSSSASPSSPSSVASPNSRASNMSPQSSASDHSASYTLQNLNLSS
STSSMGYPGMSYQQQQQHQPQHQQHHQQQHQQHHQQQHQQQIQPQQQHYYAPQLLNLDQEHLQTAS
FSYTTSSNEAFASPDADYGKPHLVILEQPVDKFRFRYQSEMHGTHGSLMGSRTEKSKKTFPTVELR
GYSGEAKVRCSLYQVDPQRRAPHSHHLVIKSGELDLIDPHDLDVGGPTGESSDGGGGGGGGGSYVA
TFQGMGIIHTAKKFIAEELYKKLRKHRLCELNREPTEREEQQMQKDATVMARTMNLNQVCLCFQAY
RVEPGTGRWVPICEPVYSNAINNMKSALTGELKICRLSTTVSGVDGGEEVFMFVEKVCKNNIKIRF

YELDEYDQEVWQEMAIFSEADVHHQYAIAFKTPPYRNKDITEPVEVHMQLYRPRDRCQSEPVLFKY
KPRSGMMVPGSSGSGSRKRLRISSGNISSEIPTVIQNDPNAGGGGGGGGVPGAGGGGGAGGGGAGS
TTRLPPLHQPFPMLANHDGSIPEGHEPSSAATINAGTATGHQQDIMSGIGSVTTISKELTKASIIQ
EILNIPTTIASDVAFDSSDFPCNSEEFNKLIQEIGNQQDLVKLETDSEAAAGDTTESVLGRAIADL
VASGDDARQGEMLRKLLALIKLFAGDVNRSRQLLASHWTAANQQQLNCLHAAIRRNDTTIACKLIE
LLDEFRLTDELLDLPNDRNETALHLAVSTNNESIVRELLKAGAKLTFCDYRGNTALHRAVVENVPA
MVRLLLRHGQAGRSRLDCTNDDGLTALQAAVYARNLKITRILIDAGASVREKDLKHGNNILHIAVD
NDSLDIVNYVLEHVNRELGLEQNNAGYTPLQLADAKSATGQGNNKLIVRELLRHFPDGLEKRTAGD
DSQEQEEDIDEDEEEEEEEEEDEEEREGEAIEQDKEQKNETVLDSINLNCDRVNIARLLEEHEPEA
EPQRKATKRSDATTATERKEPAAASAPLFDDQCMEELCELLSGSETWRALGSLLDFHPFFTVWEQG
PSPARMLLGYYEMQQLNMDRLIDMLRALELRDCIRSIDEMICRRMK

**>g376_MKK3_IndInt**
MYGSVSPDLLLIVLRFLLGAARDAVPFWRFGLVWTFTLYDDDVQVKDVFSQRSTGELSLEAHGAGP
GLKWHDQTLAATSTNRGEQEEKEGGSQQPTNNNNTNEARKVFLLSVGVSLVWAVAAAEYRLHCRAT
PPPPSPSAPKMPRKNKPIIKLKLDGDTGGGSTVPDSAEGATPRNLDKRGTLTVGERSFVVEADHLR
KLADLGRGAYGIVEKMLHQPSGTVMAVKRITVTSGMGAGGGLGGGGVQSQEQKRLLMDLDVSMRAS
DCRHTVQFYGALFREGDVWICMEVMDTSVDKFYPVVYKRPGRTIPERILGRIALAIVRALHYLHTE
LRVIHRDVKPSNVLMNRRGEVKMCDFGISGYLVDSVAKTIDAGCKPYMAPERIDPSSCSRTAGYDI
KSDVWSLGITMVEIATGRFPYATWRTPFEQLKQVVNDEPPRLPKSAAPDSEEFSAEFHAFTASCLQ
KKFQQRGNYEQLLAMEFLQRYGAGDVQADTSDMAEYVCAILDERDGVPAAEDGGGELAN

**>g20745_JUN_IndInt**
MRNNVLNSKHTNNNTMESFYEDNNAQFVPSTTTTNGVTTGNGNGLKRPATLELNLAPGKARKTRYN
ASVTMPSVLPSPDMQMLKLVSPELEKIISTNATLPTPTPSAIIFPPSATSEQQQFAKGFEDALLSI
HKKDTTSKLNTNNNNNNNNSNTSNNNNGPSANIGGAPAERLCPPTTAAPTTVVPVVGGTTTNNLAST
VLASGHNNGMSGGEITYTNLDYPGVVKEEPQATSSNQSPPVSPIDMESQERIKLERKRLRNRVAAS
KCRRRKLERISKLEERVKELKTQNSELSGVVCSLKQHIFQLKQQVIEHHNNGCTITLVGKF

**>g7769_RAC1_IndInt**
MSSGRPIKCVVVGDGTVGKTCMLISYTTDSFPGEYVPTVFDNYSAPMVVDGVQVSLGLWDTAGQED
YDRLRPLSYPQTDVFLICYSVASPSSFENVTSKWYPEIKHHCPDAPIILVGTKIDLREDRETISVL
AEQGLSALKREQGQKLANKIRAVKYMECSALTQRGLKQVFDEAVRAVLRPEPLKRRQRKCVVIEMQ
TMASGVAGNAEKETATINPREACKVNNEATKRLTNVGKS

**>g4935_MAPK_IndInt**
MSMNEGIAVVTKVVSTSVRRAPEPTAVLTREDFNAGPISLDEIEPGLWLGNASAAADMGTLEKHTI
RSILSIDSVPLPVHITDHPNLRVRHIQAADVPREDLIRHFEDSNRFIVDSLAEGRHVLVHCYFGVS
RSATIVIAYVMQKYRLNYEAAFQRVKAKRIFVMPNPGFVNQLKLYGRMAYRIDRTNERYKLFRLRL
AGDNVRKAKRLPTECMDVVKTDPGVTQESPEPYVYRCRKCRRVVASRSNLLLHKPKSATLAQSPAK
VGREPEATLEEEGTSDEAECDGQPQMNADGENVADHVTHVQTAVATGSSPSSGGGMNELAEQVRRS
SISSEHSNRSSEKDTPMCNKIFFIEPLAWMTDIYRNTQGRLYCPKCTVKLGSFNWVMATKCPCGAE
IYPAFYLVPSKTEYSTVVQNVQVTV

**>Intsfg7581_Erk_IndInt**
MAEAASSGAAAAAAAASAAGPAAGSTSATNPNAEVIRGQVFEVGPRYTNLAYIGEGAYGMVVSATD
TVTKTKVAIKKISPFEHQTYCQRTLREIKILTRFKHENRLSNDHICYFLYQILRGLKYIHSANVLH
RDLKPSNLLLNTTCDLKICDFGLARVADPEHDHTGFLTEYVATRWYRAPEIMLNSKGYTKSIDIWS

VGCILAEMLSNRPIFPGKHYLDQLNHILGVLGSPSQEDLECIINEKARSYLQSLPYKPKVPWSRLF
PNADLNALDLLGKMLTFNPHNRISPVAEEPFRIAMELDDLPKEMLKRLIFDETLRFNHNDNNLPGG
M

**>g14460_TRAF4_IndInt**
MKFPLLVRLCPGVAPSHDHLGSVSPRSKILGALVPAVCELAVRKSDVIYPDPESEKAIMGSLVFCI
HHKQGCKWSDELRKLKAHLNTCKHDAIQCPNKCGSQIPRVMMTDHLAFTCILRRAICEFCNVEFTG
IGLEEHAGTCSSEPIYCESKCGTRVLRGRMSIHRAKDCAKRLRRCPHCGREFSADTLAAHGATCPR
SPIPCPQRCDAGPFARADLDAHLRDECKALTVPCTFKEAGCRFKGPRHLLEAHLETNTSAHLSLMV
ALSGRQGQQINMLKSAMAKLSTNYTGTLLWKITDWSVKMQEAKTKDGLELVSPPFYTSQYGYKLQA
SMFLNGNGPGEGTHVSVYIKVLPGEYDALLKWPFSHSVTFTLFEQGTLGSGGGGQGGVAESFVPDP
TWENFQRPSTEPDALGFGFPRFVSHELLNRRPFVRDDTVFLRVKVDPSKIVAV

**>g488_FOS_kayak_IndInt**
MRSVRGSTLYGLGIIAFAWLAPATHHNIHKAVQGACPLAVAWWRFASFDGIGVHSGVPTRTTPTLT
PTTLKNIEQTFMEATNAQINLPYQAGFVPPSYPMEDHSQDGGVSQESLSSNSNGSWAVSGSHGYTG
DDHDSKSSASLEQPSSGTRSRRSATGTDENAKNETVTFAIGTGGSGTGARTGGRRNVGGRRPHKPS
NLTPEEEEKRRIRRERNKQAAARCRRRREDHTNELVDETDQLEKKRQSLAQEIQQLQQEKDDLEFL
LETHREHCRLQARRSPIDLKPVDLDTGGFENTGTFVLPKIKTEPEDEFAAAAAAAAAATQQHHQQP
PPNQLQEQLAIEAAEHGTISKKLKLSHAHSDSLETPTPTSSAFAPLAGVGLAGNSGNKLSATPAGS
NGSRPTRPDSLDVKSAAQYPLIARGDAAGSLAISTPSSGLFNFDSLMEGGTGLTPIAAPISFPPNR
NPLELITPTSTEPSKLCSL

**>g17088_IKBK_IndInt**
MSGLHSATFIVRGCAVNRLNIAQPAAFFRYIKTQLSVLCALLQESNRGVSLLVNVLANMIKPFEDP
PLIGDWCREKRLGNGGFGVVSLWRNKQTQQAVAIKKFHILQDRSEITDKHCERWRNEVKLMTETVQ
NENIVRTVNVQPTSFIQELLRSSANGLPVLCMEYCEGGDLRRVLNRVENCSGLREQDVRDVLRSLR
NAVAYLHSLKITHRDIKPENLVLKQQGERFIYKADLGYAKALDKQSLNASLVGTVEYIAPDLIYCD
RYNCSVDYWSMGVIGYEIITGVRPFIPHAPITRWMMHVQQKKSADIAITEDNRENYTYHTDIFPEN
HISDCLRKELESWFRLALEWNPKKRGYVPYTKAEPSLANGENGTKGVKQTEDNKPSTVLKIFSLLD
QILEKRILVLFSLYDCRWIDLEVTPETGMETLRDHVYRVTGIPVDDIEFVLPLEQKQPAVGNDTRP
YDLYLPDFYGKPMVYVVHRGSRDSIVQRDLKPRIPKSITDVFQNIKVKLKPHMLRQFIANSYYFIA
QEQRLYGQVLDGIRNYGLMLNDNIARRKDEIGRMNKIVYAILGGVEYHKLTVCHARDALNVERRIP
SATFEMASKRWIENGARIESNVRKLAEMADTITKRYESVLKRSRDALRHALLHPQLDQHDTFGLRN
VECRYEQTRARLMEKILNEKSHMDMSQAVYECLKQRDVLLRELGFLELQQQILDVRREIQEIEKVV
CKVIDVTEKYKRDLARLKLEHQDEVWKMLSDYGRSAAAGSCENGVVHLDNILPLGVAHTDPPVINK
PNFLVGGPTTPQLQSTVSNESFDANQVCCLGYTDPNVEDLIAANETLIHTTTDLLSNSFSLLKLAD
GAE

*Supplementary Text 2 : FASTA sequences of the genes from the IndInt strain involved in eiger mediated TNF pathway.*
